# Genetic determinants of metabolic–inflammatory dysregulation and machine learning prediction of COVID-19

**DOI:** 10.3389/fcimb.2026.1874596

**Published:** 2026-08-05

**Authors:** Larysa Sydorchuk, Maksym Sokolenko, Ruslan Sydorchuk, Alina Sokolenko, Iryna Kamyshna, Ludmila Sokolenko, Petro Moroz, Andrii Sydorchuk, Oleksandr Sokolenko, Iryna Halabitska, Pavlo Petakh, Oleksandr Kamyshnyi

**Affiliations:** 1 Department of Family Medicine, Bukovinian State Medical University, Chernivtsi, Ukraine; 2 Department of Infectious Diseases and Epidemiology, Bukovinian State Medical University, Chernivtsi, Ukraine; 3 Department of General Surgery, Bukovinian State Medical University, Chernivtsi, Ukraine; 4 Department of Medical Rehabilitation, I. Horbachevsky Ternopil National Medical University, Ternopil, Ukraine; 5 Department of Medical and Biological Fundamentals of Physical Culture, Pavlo Tychyna Uman State Pedagogical University, Uman, Ukraine; 6 Department of Surgery No1, Bukovinian State Medical University, Chernivtsi, Ukraine; 7 Donauklinik, Neu Ulm, Germany; 8 Bukovinian State Medical University, Chernivtsi, Ukraine; 9 Department of Therapy and Family Medicine, I. Horbachevsky Ternopil National Medical University, Ternopil, Ukraine; 10 Department of Biochemistry and Pharmacology, Uzhhorod National University, Uzhhorod, Ukraine; 11 Department of Microbiology, Virology, and Immunology, I. Horbachevsky Ternopil National Medical University, Ternopil, Ukraine

**Keywords:** COVID-19, FGB (rs1800790), genes polymorphism, inflammation, machine-learning models, metabolism, NOS3 (rs2070744), TMPRSS2 (rs12329760)

## Abstract

The aim of this study was to identify genetic variations that influence the metabolic-inflammatory profile of coronavirus disease and to evaluate the performance of modern machine learning models for the classification and prediction of COVID-19 severity. Real-time polymerase chain reaction was used to genotype the polymorphism of FGB (rs1800790), NOS3 (rs2070744) and TMPRSS2 (rs12329760) genes. Model performance was assessed using Accuracy and AUC-ROC, with a focus on maximizing AUC-ROC to ensure optimal discrimination, and SHAP analysis. Optimal hyperparameters for each model were defined as those yielding the highest mean AUC-ROC value during 5-fold cross-validation. COVID-19 severity is strongly associated with a pronounced pro-inflammatory and pro-endothelial activation profile, characterized by significantly elevated transmembrane serine protease 2 (TMPRSS2), endothelin-1 (ET-1), interleukin-6 (IL-6) and procalcitonin (PCT) levels, together with marked metabolic dysregulation. Genetic variations contribute to inter-individual differences in biomarker expression, with specific allelic variants modulating inflammatory intensity and endothelial dysfunction. In particular, the FGB rs1800790 A-allele and eNOS rs2070744 ТТ-genotype are associated with a more pronounced inflammatory response and endothelial dysfunction, while rs12329760 TMPRSS2 variants show weaker but detectable modulatory effects with higher transmembrane serine protease 2 value in T-allele moderate-severe COVID-19 patients. Ensemble methods such as ExtraTreesClassifier (Accuracy: 0.974 ± 0.022) and RandomForestClassifier (Accuracy: 0.960 ± 0.035) demonstrated the highest ROC curves approaching, confirming their encouraging performance in predicting COVID-19 severity. Simpler models, including BernoulliNB (Accuracy: 0.956 ± 0.037) and DecisionTreeClassifier (Accuracy: 0.938 ± 0.043), also showed high classification quality. Analysis of misclassification patterns revealed that ExtraTreesClassifier, HistGradientBoostingClassifier, BaggingClassifier, and GradientBoostingClassifier made no errors across any class. The poorest performance was observed with LinearDiscriminantAnalysis, which generated 11 misclassifications, followed by CalibratedClassifierCV, and LogisticRegressionCV. External validation of the obtained results in larger multicentre cohorts is essential before clinical implementation.

## 1 Introduction

Emergence of severe acute respiratory syndrome coronavirus 2 (SARS-CoV-2) causing coronavirus disease 2019 (COVID-19) represents a major global health challenge due to its widespread transmission and severity (Lai et al., 2020; El-Kassas et al., 2023; Kolesnyk et al., 2024). COVID-19 affects upper and lower airways with significant systemic manifestations. A comprehensive understanding of the factors influencing COVID-19 susceptibility and severity is crucial for effective prevention strategies and improved clinical management (Schoeni et al., 2021; Kolesnyk et al., 2024). Based on current evidence, major risk factors for the severity and mortality of COVID-19 include older age, male sex, and ethnicity, and comorbidities such as cardiovascular disease, obesity, chronic respiratory disease, diabetes, cerebrovascular disease, chronic renal failure (De Simone et al., 2022; Martono et al., 2023; Zhang et al., 2023; Sokolenko et al., 2024). Predictors of severe COVID-19 include multiple inflammatory and acute-phase biomarkers, including C-reactive protein (CRP), procalcitonin (PCT), leukocyte alterations, lymphopenia, and interleukin 6 (IL-6) (Wolff et al., 2021; Sokolenko et al., 2025; Stec-Martyna et al., 2025; Li et al., 2026). Severe inflammation causes D-dimer elevation, reflecting hypercoagulable and thrombotic states, whereas other coagulation markers showed no reliable statistical or clinical differences (Herold et al., 2020; Lippi and Plebani, 2020; Wang et al., 2022; He and Ding, 2024). Markers indicating cardiac damage, such as cardiac troponin I or T, and N terminal pro B type natriuretic peptide are also associated with severe COVID-19 course and mortality (Tang et al., 2020; Wang et al., 2020; Khan et al., 2021).

At the same time, metabolic and inflammatory factors influence the coronavirus disease severity likewise. Obesity, type 2 diabetes, and dyslipidaemia are typically accompanied by sustained metabolic dysregulation and inflammation, which can modulate immune responses to SARS-CoV-2 (Semianiv et al., 2021; Esmaeilzadeh et al., 2022; Petakh et al., 2022; Nour and Altintaş, 2023; Klaudel et al., 2025). On the other hand, increasing evidence indicates that disease severity is driven by the complex interplay of several biological pathways, including viral entry mechanisms, endothelial dysfunction, coagulation abnormalities, and dysregulated inflammatory responses. SARS-CoV-2 infection triggers activation of host proteases and immune signalling cascades, which may lead to excessive cytokine production, endothelial injury, microvascular thrombosis, and impaired tissue perfusion. These processes contribute to the development of thromboinflammation, a hallmark of severe COVID-19 (Jabczyk et al., 2021; Powell et al., 2021; Rahnavard et al., 2022; Choudhri et al., 2023; Zsichla and Müller, 2023; Li et al., 2026). Host genetic variability may further modulate individual susceptibility to these pathogenic mechanisms. Genetic variants affecting viral entry, endothelial homeostasis, and coagulation pathways have been proposed as contributors to inter-individual differences in disease severity and clinical outcomes. In parallel, circulating biomarkers reflecting endothelial activation, dysmetabolic and inflammatory burden, and coagulation status have emerged as important indicators of disease progression. Understanding the interaction between genetic predisposition and biological response pathways may improve risk stratification and facilitate the development of personalized predictive approaches.

On the other hand, timely identification of patients with a high probability of developing critical conditions is of fundamental importance for optimizing medical care. Early risk stratification allows not only to identify patients who require urgent intervention, but also to rationally allocate health care resources, which can ultimately contribute to reducing mortality (Bosello et al., 2019; Gao et al., 2020; Li et al., 2026). In this context, artificial intelligence (AI) is considered a promising tool that can provide effective approaches to analysing large volumes of medical data and solving key problems of the pandemic (Bajwa et al., 2021; Buttia et al., 2023; Gawande et al., 2025; Rabbani et al., 2025).

AI based methods, particular predictive analytics, intelligent diagnostic systems, risk assessment, clinical decision support and computer-aided drug discovery, demonstrate significant potential in increasing the accuracy of detection, prognosis and treatment of COVID-19 (Fu et al., 2022; Abbas et al., 2025; Fahim et al., 2025; Mendhi et al., 2025). In particular, machine learning (ML) allows algorithms to go beyond strictly predefined rules and instead identify patterns in data (Koteluk et al., 2021; Rattan et al., 2022; De Simone et al., 2024). In this way, predictive models are built through the analysis of large and complex datasets rather than through manually specified instructions (Kaur et al., 2021; Eze et al., 2024; Jamarani et al., 2024; Rani et al., 2025).

In practice, ML has increasingly been used for risk prediction and stratification, and its advantages become especially evident when dealing with complex, nonlinear relationships, heterogeneous variables, and large-scale data (Shah et al., 2025; Eini et al., 2026; Kasundra and Ghada, 2026; Tang et al., 2026). At the same time, a number of studies have shown that ML models can effectively identify higher risk patients of severe COVID-19 at early stages, predict outcomes for intensive care, mechanical ventilation, and the likelihood of mortality based on clinical, laboratory, coagulation, and genomic features (Rahmani et al., 2021; Li et al., 2022; Liu et al., 2025). However, the findings across studies are not always consistent. In some cases, the reported results differ quite noticeably, which can be attributed to variations in sample size, population characteristics, and data quality. Variations in feature selection, model tuning, and performance assessment methods may partly explain the observed differences. In addition, many studies still face methodological limitations: reproducibility is not always clearly addressed, systematic comparisons between models are often lacking, and external validation remains limited or absent in a substantial proportion (Tulu et al., 2023; Sethi et al., 2024).

Against this background, the present study aims to identify whether genetic variants representing coagulation, endothelial, and viral-entry pathways, together with circulating inflammatory and endothelial biomarkers, are associated with COVID-19 severity and can improve machine-learning-based risk stratification. Candidate genetic variants were selected based on their established involvement in key biological pathways implicated in COVID-19 pathogenesis, including coagulation (FGB rs1800790), endothelial function (NOS3 rs2070744), and SARS-CoV-2 host-cell entry (TMPRSS2 rs12329760). These polymorphisms have previously been associated with inter-individual variability in inflammatory responses, endothelial dysfunction, thrombotic risk, and disease severity. Moreover, in our previous study (Sokolenko et al., 2024; Sokolenko et al., 2025), these variants demonstrated significant associations with COVID-19 severity and inflammatory-endothelial biomarkers, providing the biological rationale for their inclusion in the present integrated ML analysis. The analysis is based on Accuracy and AUC-ROC metrics, with particular attention given to AUC-ROC as a more informative indicator of model performance and SHAP analysis. The present exploratory analysis was designed to compare the internal classification performance of different machine-learning algorithms and to identify the variables contributing most strongly to severity classification. The findings may provide a basis for the future development of interpretable risk-stratification tools, subject to confirmation in independent external cohorts.

## 2 Materials and methods

### 2.1 Study design and participants

This cohort study included 257 patients with laboratory-confirmed COVID-19, among whom 197 exhibited moderate-to-severe disease and 60 had a mild clinical course. COVID-19 diagnosis in all participants was established using polymerase chain reaction (PCR) testing. The mild COVID-19 group (n = 60) consisted of non-hospitalized individuals managed in primary care settings. Mild disease was defined based on the following clinical criteria: body temperature ≤38 °C, presence of rhinitis and dry cough without evidence of respiratory insufficiency (e.g., dyspnoea, tachypnoea, or haemoptysis), absence of gastrointestinal manifestations (nausea, vomiting, and/or diarrhoea), and no neurological or cognitive impairment (including altered consciousness or lethargy). Within this cohort, 40.0% (n = 24) were male and 60.0% (n = 36) female, with a mean age of 56.77 ± 5.67 years. Vaccinated individuals accounted for 66.7% (n = 40), whereas 33.3% (n = 20) were unvaccinated; 20 participants were active smokers. No statistically significant differences in age or sex distribution were observed compared to the moderate-to-severe group (p > 0.05).

The moderate-to-severe group (n = 197) comprised hospitalized patients treated at the Infectious Disease Department of Uman Central City Hospital for community-acquired COVID-19 pneumonia. Among them, 21.97% (n = 55) were classified as having moderate disease and 78.03% (n = 142) as severe. Moderate COVID-19 was defined by the presence of fever, dry cough, dyspnoea, and tachypnoea (20–30 breaths/min) without features of severe pneumonia, including oxygen saturation (SpO_2_) ≥90%. Severe disease was diagnosed when these symptoms were accompanied by at least one of the following: respiratory rate ≥30 breaths/min, oxygen saturation ≤90%, PaO_2_/FiO_2_ ratio <300, or pulmonary infiltrates affecting more than 50% of lung fields. Pneumonia was confirmed in all cases using imaging modalities such as computed tomography (CT) or chest radiography. Diagnostic procedures, laboratory assessments, and therapeutic interventions were conducted in accordance with Ukrainian Ministry of Health guidelines for COVID-19 management, as well as WHO, CDC, and international standards (Ministry of Health of U, 2020a; Ministry of Health of U, 2020b; Centers for Disease C Prevention, 2023).

Comparative analysis within the moderate-to-severe cohort revealed no statistically significant differences between patients with moderate and severe disease in terms of age, systolic and diastolic blood pressure, or body mass index, although a tendency toward less favourable values was observed in the severe subgroup. The overall sex distribution was balanced (97 males, 100 females); however, females were more prevalent among severe cases, whereas males predominated in moderate cases by 17.45% (χ² = 4.83; p = 0.028). In both subgroups, the proportion of unvaccinated individuals was approximately twice that of vaccinated patients, with no significant intergroup difference (χ² = 0.4; p = 0.527).

The study was conducted in accordance with international ethical standards, including ICH/GCP guidelines, the Declaration of Helsinki (1964), and the Council of Europe Convention on Human Rights and Biomedicine (1997), as well as applicable national regulations. Ethical approval was obtained from the Ethics Committee of Bukovinian State Medical University (Protocol No. 2, October 2025). Written informed consent was secured from all participants prior to study inclusion.

### 2.2 Clinical and laboratory assessments

A comprehensive laboratory examination was performed, including the determination of oxygen saturation (SpO_2_, %), transmembrane serine protease 2 (TMPRSS2), endothelin-1 (ET-1), interleukin-6 (IL-6), procalcitonin (PCT).

SpO_2_ was measured using a pulse oximeter (Gamma Oxy Scan, SHENZHEN HOMED MEDICAL DEVICE, China).

The serum level of TMPRSS2, IL-6, ET-1 and PCT were determined by Enzyme Linked-Immuno-Sorbent Assay (ELISA) and Electrochemiluminescence Immunoassay (ECLIA) according to the Manufacturer’s Guidelines with highly sensitive TMPRSS2 ELISA KIT^®^ (MyBioSource, Inc. P.O. Box 153308 San Diego, CA 92195-3308, USA), Human Interleukin-6 ELISA KIT^®^ (Elabscience Biotechnology Inc.Houston, Texas, 77079, USA), Human ET-1 ELISA KIT^®^ (Elabscience Biotechnology Inc.Houston, Texas, 77079, USA), PCT ECLIA KIT^®^ (Roche Elecsys^®^ PCT, France) on “MAGLUMI 1000 Plus” device (“SNIBE”, China).

ELISA principles for TMPRSS2, IL-6 and ET-1 evaluation: traditional microplate format using capture and detection antibodies. After sample incubation and washing, a substrate for the enzyme-conjugated secondary antibody yields a colorimetric readout proportional to reliable determined parameter (TMPRSS2, IL-6, ET-1). Sensitivity: ~0.1 ng/mL.

ECLIA principles for PCT determination: sandwich assay – one biotinylated antibody and one ruthenium-labelled antibody bind PCT. Streptavidin-coated microparticles capture the complex; electrical stimulation triggers light emission from the ruthenium label, quantified by a photomultiplier. Sensitivity: ~0.02 ng/mL.

D-dimer was determined by Electrochemiluminescence Immunoassay (ECLIA) with highly sensitive Elecsys D-Dimer ECLIA KIT^®^ (Roche Diagnostics^®^, France) on “MAGLUMI 1000 Plus” device (“SNIBE”, China).

Fibrinogen was determined by the Clauss method with quantitative FBG reagent Dia-FIB^®^ (Diagon GmbH, Germany) on the semi-automated coagulation analyser DIAGON CoagM, manufactured by Diagon Ltd. (Hungary). The method of Clauss measures the clotting time after adding a high concentration of thrombin to diluted plasma. The Fibrinogen concentration in the plasma is inversely proportional to the clotting time. Sensitivity ~0.05 g/L.

Biochemical parameters were evaluated on biochemical automatic analyser “ACCENT 200” (CORMAY, Poland).

Depression was assessed with the validated PHQ-9 questionnaire (Patient Health Questionnaire-9) with cut-off value >4 score. The psychosomatic contribution of depression to disease (COVID-19) progression was evaluated in our former publication (Sydorchuk et al., 2025).

### 2.3 Genotyping of FGB, NOS3, and TMPRSS2 polymorphisms

Genotyping of the FGB (rs1800790), NOS3 (rs2070744), and TMPRSS2 (rs12329760) genes was performed for 72 patients with moderate-severe COVID-19 (study group) and for 48 patients with mild COVID-19 course (control group).

For the isolation of genomic DNA, peripheral blood leukocytes were used using a commercial kit (Thermo Scientific™ GeneJET™ Whole Blood Genomic DNA Purification Mini Kit). 200 μL of whole blood from each participant was digested with proteinase K followed by the addition of lysis buffer. The next steps included washing and elution of the purified DNA.

Real-time polymerase chain reaction (RT-PCR) was used to genotype the polymorphism of the FGB (rs1800790), NOS3 (rs2070744), and TMPRSS2 (rs12329760) genes. For this purpose, the CFX96™ real-time PCR detection system (Bio-Rad Laboratories, Inc., USA) was used. Specific TaqMan™ kits were used for each target SNP. Genotyping was performed using TaqMan^®^ probes and TaqMan^®^ Genotyping Master Mix (4371355) in combination with the CFX96™ Real-Time PCR Detection System and was described in our former publication (Klaudel et al., 2025). The PCR protocol was strictly followed the manufacturer’s instructions (Applied Biosystems, USA). The TaqMan^®^ Genotyping Master Mix includes AmpliTaq Gold^®^ DNA polymerase, dNTPs, ROX™ reference dye, and optimized reaction buffers. For the identification of gene alleles, TaqMan^®^ probes were used, which are allele-specific oligonucleotides with reporter dyes (VIC^®^ for allele 1 and 6-FAM™ for allele 2) attached to the 5’ end and a non-fluorescent quencher (NFQ) at the 3’ end. Genomic DNA (10 μL) was amplified in a reaction mixture containing primers, probes, master mix, and target DNA. For genotyping, allele discrimination based on relative fluorescence units (RFUs) was used using CFX-Manager™ software. PCR cycle conditions were as follows: Initial denaturation: 95 °C for 10 minutes; amplification cycles (49 cycles); denaturation: 95 °C for 15 seconds; annealing: 60 °C for 1:10 minutes; final melting curve analysis: Increase in temperature to 95 °C. Genotype determination was based on melting curve analysis using CFX96™ Real-Time PCR Basic software (Bio-Rad Laboratories, Inc., USA).

The genotypes distributions of FGB (rs1800790), NOS3 (rs2070744) and TMPRSS2 (rs12329760) genes were consistent with Hardy-Weinberg equilibrium (all P > 0.05). These findings were reported in our former publication (Sokolenko et al., 2025).

### 2.4 Dataset preparation and preliminary model screening

The primary dataset was 257 patients (60 – mild, 55 – moderate and 142 – severe). For the purposes of machine learning analysis, a final dataset comprising 226 observations (with severity encoded as 0 = mild, 1 = moderate, 2 = severe, and class sizes of 142, 54, and 30, respectively) and 68 variables was constructed; records from 31 patients were excluded due to incompleteness detected by automated classification algorithms.

The dataset incorporated demographic characteristics (age, sex), genetic variables (polymorphisms in FGB, NOS3, and TMPRSS2 genes), biochemical markers (including IL-6, endothelin-1, D-dimer, fibrinogen, among others), and clinical parameters (blood pressure, body mass index (BMI), comorbid conditions). The dependent variable was disease severity, encoded as a categorical outcome (0 = mild, 1 = moderate, 2 = severe). The utility of routine laboratory biomarkers for COVID-19 risk stratification has been previously established. Categorical variables comprised sex, vaccine type, genotypes of FGB, NOS3, and TMPRSS2, as well as disease severity. Missing data (<1%) were observed only for several binary encoded clinical variables (Fam.Pulmo, Fam.Cardio, Fam.Collagenosis, MI, Compl.Nephro, Compl.Gastro, and PAD). Given the very low proportion of missing values, these were imputed using mode substitution for binary variables. COVID-19 severity categories and genotype variables contained no missing values and therefore were not subjected to imputation.

It should be noted that the dichotomous classification (mild vs. moderate-to-severe) was applied exclusively in the genetic association analyses because genotyping was performed in a predefined subset of patients (n = 120). In contrast, all machine-learning analyses were conducted using the complete dataset with three severity categories (mild, moderate, and severe) encoded as the prediction outcome (n = 226).

To compare the performance of commonly used machine-learning classifiers for COVID-19 severity classification, a broad set of algorithms available in the Scikit-learn library was initially evaluated using default hyperparameter settings. The outcome comprised three severity categories: mild, moderate, and severe COVID-19.

Model performance was assessed using stratified 5-fold cross-validation. In each cross-validation iteration, four folds were used for model training and the remaining fold was used for validation, so that each fold served as the validation set once. Stratification preserved the proportions of the three severity categories within each fold.

For the multiclass outcome, ROC-AUC was calculated using the One-vs-Rest approach, and the overall ROC-AUC was weighted according to class size. Accuracy was reported together with precision, recall, and F1-score. No explicit resampling, oversampling, undersampling, or class weighting was applied. Consequently, the reported weighted performance estimates may have been influenced by the predominance of the largest severity class and should be interpreted cautiously.

### 2.5 Model optimization, internal validation, and interpretability

To identify predictors of COVID-19 severity and evaluate the combined contribution of genetic, biochemical, inflammatory, and endothelial biomarkers, supervised machine learning (ML) models were developed using the complete dataset comprising 226 observations and 68 variables.

Prior to model training, data quality was assessed, and missing values were handled using median imputation for continuous variables and mode imputation for categorical variables. Continuous variables were standardized when required by the algorithm. Categorical variables were encoded using binary or ordinal encoding as appropriate. The percentage of missing values was low (<1%) for each variable.

The dataset was randomly partitioned into training and testing subsets using stratified sampling to preserve the proportion of mild and moderate–severe COVID-19 cases. Model development was performed exclusively on the training dataset, while the testing dataset was reserved for final performance assessment.

To minimize overfitting and improve model generalizability, repeated stratified k-fold cross-validation was applied during model training. Specifically, the training set was evaluated using 5-fold cross-validation repeated five times. In each iteration, nine folds were used for model training and one fold for validation, ensuring that every observation was included in both training and validation subsets across repetitions.

Several supervised learning algorithms were evaluated, including Logistic Regression, Decision Tree, Random Forest, Extra Trees, Gradient Boosting, BernoulliNB, Hist Gradient Boosting, Calibrated Classifier, Bagging Classifier and Linear Discriminant Analysis. Hyperparameter optimization was performed using GridSearchCV with cross-validation to identify the optimal model configuration for each classifier.

To minimize the risk of information leakage and obtain an unbiased estimate of model performance, hyperparameter tuning was conducted exclusively within the training data of each cross-validation fold using GridSearchCV to optimize the most relevant model parameters. The optimal parameter combination identified during training was then applied to the corresponding validation fold for performance assessment. Consequently, validation data were not involved in either model training or hyperparameter optimization. Owing to the relatively small sample size, no independent hold-out test set was established; therefore, all reported performance measures represent estimates derived from internal cross-validation. The independent test dataset was not used during feature selection, hyperparameter optimization, or model development.

Model performance was assessed using multiple complementary metrics, including accuracy, area under the receiver operating characteristic curve (ROC-AUC), precision, recall (sensitivity), and F1-score. These metrics were calculated from predictions generated on the independent test dataset. ROC curves were constructed to evaluate discrimination ability across classification thresholds.

To improve interpretability and identify the most influential predictors contributing to model performance, feature importance analysis was performed using both impurity-based importance measures and SHapley Additive exPlanations (SHAP). SHAP summary plots were generated to quantify the direction and magnitude of individual feature contributions to model predictions.

The final predictive models were internally validated; however, external validation in an independent cohort was not available and therefore remains a necessary step before clinical implementation.

### 2.6 Correlation and multicollinearity analysis

To explore relationships among variables and address potential multicollinearity, thereby minimizing redundancy prior to machine learning modelling, a comprehensive correlation analysis was performed. Pearson’s correlation coefficient was applied to continuous variables, while Cramér’s V (derived from the chi-square statistic) was used for categorical variables. For associations between continuous and categorical variables, the correlation ratio (η) was employed to estimate the proportion of variance in the continuous variable explained by the categorical factor.


$${\text{η}}^{2}=\frac{SS_{between}}{SS_{\text{total}}}$$

Here, *SS_between_* – denotes the sum of squares between groups (defined by categorical variables), *SS_total_* – sum of squares between groups (determined by categories), represents the total sum of squares. The value of η ranges between 0 and 1, with higher values indicating stronger dependence.

### 2.7 Statistical analysis of clinical and genetic data

Statistically, the results were processed in accordance with contemporary requirements, using the Statistica 13.0 program (StatSoft Inc, USA). Pearson’s criterion (χ2) was used for the genotype distribution comparison. The reliability of data for independent samples with an array distribution close to normal was calculated by the Student’s t-test, and in case of uneven distribution – by the Wilcoxon-Mann-Whitney U test. Differences were considered significant at p<0.05.

## 3 Results

Moderate-severe COVID-19 was associated with significantly elevated TMPRSS2, ET-1, IL-6, and PCT levels across all rs1800790 (FGB), rs2070744 (eNOS), and rs12329760 (TMPRSS2) genotypes compared with mild disease course (all p ≤ 0.004) (**Table 1**, **2**, **3**).

**Table 1:** Inflammatory activity markers and transmembrane serine protease 2 values in the examined subjects depending on the rs1800790 genotypes (FGB gene).

Data	Genotypes	Mild COVID-19 (control)	Moderate-severe COVID-19
TMPRSS2, ng/mL	GG-genotype	1.60 ± 0.06	2.61 ± 0.20 р<0.001
	A-allele	1.88 ± 0.13 р_GG_=0.047	2.63 ± 0.17 р<0.001
ET-1, pg/mL	GG-genotype	3.50 ± 0.24	10.84 ± 2.62 р=0.004
	A-allele	4.20 ± 0.58	13.66 ± 3.07 р=0.004
IL-6, pg/mL	GG-genotype	5.32 ± 0.39	63.49 ± 6.77 р<0.001
	A-allele	8.62 ± 1.43 р_GG_=0.035	72.92 ± 7.40 р<0.001
PCT, ng/mL	GG-genotype	0.10 ± 0.001	0.32 ± 0.08 р<0.001
	A-allele	0.10 ± 0.001	0.25 ± 0.05 р<0.001

TMPRSS2, transmembrane serine protease 2; ET-1, endothelin-1; IL-6, interleukin-6; PCT, procalcitonin; p, significance of differences with the control group (mild COVID-19), corresponding genotype; pGG, significance of differences with GG-genotype carriers in each group separately.

**Table 2:** Inflammatory activity markers and transmembrane serine protease 2 values in the examined subjects depending on the rs2070744 (eNOS gene).

Data	Genotypes	Mild COVID-19 (control)	Moderate-severe COVID-19
TMPRSS2, ng/mL	TT-genotype	1.79 ± 0.10	2.62 ± 0.28 р<0.001
	TC-genotype	1.83 ± 0.11	2.59 ± 0.20 р<0.001
	CC-genotype		2.67 ± 0.15 р<0.001
ET-1, pg/mL	TT-genotype	5.14 ± 0.64	16.34 ± 3.26 р<0.001
	TC-genotype	3.36 ± 0.33 р_TT_=0.034	10.17 ± 2.64 р<0.001; р_TT_=0.049
	CC-genotype		8.54 ± 1.79 р<0.001; р_TT_=0.034
IL-6, pg/mL	TT-genotype	10.11 ± 2.25	62.90 ± 12.44 р<0.001
	TC-genotype	6.41 ± 0.61 р_TT_=0.048	77.27 ± 10.13 р<0.001
	CC-genotype		60.28 ± 11.0 р<0.001
PCT, ng/mL	TT-genotype	0.1 ± 0.001	0.35 ± 0.06 р<0.001
	TC-genotype	0.1 ± 0.001	0.23 ± 0.05 р<0.001; р_TT_=0.048
	CC-genotype		0.25 ± 0.04 р<0.001; р_TT_=0.05

TMPRSS2, transmembrane serine protease 2; ET-1, endothelin-1; IL-6, interleukin-6; PCT, procalcitonin; p, significance of differences with the control group (mild COVID-19), corresponding genotype; pTT, significance of differences with TT-genotype carriers in each group separately.

**Table 3:** Inflammatory activity markers and transmembrane serine protease 2 values in the examined subjects depending on the rs12329760 (TMPRSS2 gene).

Data	Genotypes	Mild COVID-19	Moderate-severe COVID-19
TMPRSS2, ng/mL	CC-genotype	1.79 ± 0.14	2.46 ± 0.14 р<0.001
	T-allele	1.83 ± 0.09	2.80 ± 0.17 р<0.001; р_CC_=0.048
ET-1, pg/mL	CC-genotype	4.71 ± 0.70	12.55 ± 2.56 р<0.001
	T-allele	3.61 ± 0.36	11.84 ± 2.85 р<0.001
IL-6, pg/mL	CC-genotype	7.04 ± 0.57	72.41 ± 13.0 р<0.001
	T-allele	8.25 ± 0.87	62.45 ± 9.67 р<0.001
PCT, ng/mL	CC-genotype	0.10 ± 0.001	0.29 ± 0.07 р<0.001
	T-allele	0.10 ± 0.001	0.27 ± 0.06 р<0.001

TMPRSS2, transmembrane serine protease 2; ET-1, endothelin-1; IL-6, interleukin-6; PCT, procalcitonin; p, significance of differences with the control group (mild COVID-19), corresponding genotype; pCC, significance of differences with CC-genotype carriers in each group separately.

Among A-allele carriers of the FGB rs1800790 gene, TMPRSS2 and IL-6 values were significantly higher than in GG-genotype patients with mild COVID-19 course by 17.5% and 62.03% (p=0.047 and p=0.035), respectively (**Table 1**). These findings suggest that disease severity is associated with enhanced inflammatory and endothelial activation, while the A-allele may contribute to a stronger pro-inflammatory profile.

Moderate-severe COVID-19 was associated with pronounce metabolic disturbances compared with mild disease, including higher plasma glucose, total cholesterol (TC), low density lipoprotein cholesterol (LDL-C), triglycerides (TG), and atherogenic index (AI), together with lower high density lipoprotein cholesterol (HDL-C) (all p < 0.001) for all evaluated genes (rs1800790, rs2070744, rs12329760) (**Table 4**, **5**, **6**). In the mild COVID-19 group, A-allele carriers of the FGB rs1800790 had higher glucose, TC, and triglycerides levels than GG-genotype carriers by 6.47-14.17%, (p = 0.041-0.05). These findings indicate that severe COVID-19 is linked to pronounced dysmetabolism, while the A-allele may predispose to a less favourable metabolic pattern (**Table 4**).

**Table 4:** Metabolic markers depending on the rs1800790 (FGB gene genotypes).

Data	Genotypes	Mild COVID-19 (control)	Moderate-severe COVID-19
Plasma glucose, mmol/L	GG-genotype	4.88 ± 0.10	6.95 ± 0.60 р<0.001
	A-allele	5.23 ± 0.15 р_GG_=0.041	6.24 ± 0.48 р=0.003
TC, mmol/L	GG-genotype	4.33 ± 0.10	6.51 ± 0.30 р<0.001
	A-allele	4.61 ± 0.15 р_GG_=0.05	6.29 ± 0.28 р<0.001
HDL-C, mmol/L	GG-genotype	1.74 ± 0.06	0.90 ± 0.08 р<0.001
	A-allele	1.87 ± 0.07	0.95 ± 0.07 р<0.001
LDL-C, mmol/L	GG-genotype	2.42 ± 0.05	5.32 ± 0.36 р<0.001
	A-allele	2.42 ± 0.07	5.12 ± 0.34 р<0.001
TG, mmol/L	GG-genotype	1.27 ± 0.06	3.32 ± 0.30 р<0.001
	A-allele	1.45 ± 0.10 р_GG_=0.049	3.43 ± 0.23 р<0.001
AI, U	GG-genotype	1.55 ± 0.09	7.87 ± 1.23 р<0.001
	A-allele	1.49 ± 0.07	6.63 ± 0.86 р<0.001

TC, total cholesterol; HDL-C/LDL-C, high/low density lipoprotein cholesterol; TG, triacylglycerol; AI, atherogenic index; p: significance of differences with the control group (mild COVID-19), corresponding genotype; pGG: significance of differences with GG-genotype carriers in each group separately.

**Table 5:** Metabolic markers depending on the rs2070744 (eNOS gene genotypes). .

Data	Genotypes	Mild COVID-19 (control)	Moderate-severe COVID-19
Plasma glucose, mmol/L	TT-genotype	5.23 ± 0.14	6.26 ± 0.49 р=0.01
	TC-genotype	5.05 ± 0.15	6.70 ± 0.58 р<0.001
	CC-genotype		7.04 ± 0.64 р<0.001
TC, mmol/L	TT-genotype	4.45 ± 0.09	6.53 ± 0.31 р<0.001
	TC-genotype	4.58 ± 0.20	6.33 ± 0.36 р<0.001
	CC-genotype		6.31 ± 0.30 р<0.001
TG, mmol/L	TT-genotype	1.35 ± 0.10	3.25 ± 0.22 р<0.001
	TC-genotype	1.42 ± 0.08	3.41 ± 0.31 р<0.001
	CC-genotype		3.56 ± 0.25 р<0.001
HDL-C, mmol/L	TT-genotype	1.81 ± 0.05	0.94 ± 0.08 р<0.001
	TC-genotype	1.85 ± 0.09	0.93 ± 0.08 р<0.001
	CC-genotype		0.88 ± 0.07 р<0.001
LDL-C, mmol/L	TT-genotype	2.40 ± 0.05	5.33 ± 0.34 р<0.001
	TC-genotype	2.44 ± 0.07	5.17 ± 0.35 р<0.001
	CC-genotype		5.09 ± 0.36 р<0.001
AI, U	TT-genotype	1.49 ± 0.07	7.79 ± 1.33 р<0.001
	TC-genotype	1.52 ± 0.08	6.91 ± 0.94 р<0.001
	CC-genotype		6.89 ± 0.75 р<0.001

TC, total cholesterol; TG, triacylglycerol; HDL-C/LDL-C, high/low density lipoprotein cholesterol; AI – atherogenic index; p: significance of differences with the control group (mild COVID-19), corresponding genotype.

**Table 6:** Metabolic markers depending on the rs12329760 (TMPRSS2 gene genotypes).

Data	Genotypes	Mild COVID-19	Moderate-severe COVID-19
Plasma glucose, mmol/L	CC-genotype	5.25 ± 0.12	6.75 ± 0.61 р<0.001
	T-allele	5.0 ± 0.16	6.41 ± 0.52 р<0.001
TC, mmol/L	CC-genotype	4.59 ± 0.14	6.46 ± 0.29 р<0.001
	T-allele	4.47 ± 0.15	6.33 ± 0.28 р<0.001
HDL-C, mmol/L	CC-genotype	1.90 ± 0.05	0.93 ± 0.08 р<0.001
	T-allele	1.76 ± 0.09 р_CC_=0.05	0.91 ± 0.07 р<0.001
LDL-C, mmol/L	CC-genotype	2.39 ± 0.07	5.29 ± 0.37 р<0.001
	T-allele	2.46 ± 0.05	5.13 ± 0.35 р<0.001
TG, mmol/L	CC-genotype	1.51 ± 0.09	3.48 ± 0.27 р<0.001
	T-allele	1.28 ± 0.09 р_CC_=0.034	3.25 ± 0.25 р<0.001
AI, U	CC-genotype	1.42 ± 0.05	7.05 ± 0.95 р<0.001
	T-allele	1.59 ± 0.09 р_CC_=0.048	7.50 ± 1.20 р<0.001

TC, total cholesterol; HDL-C/LDL-C, high/low density lipoprotein cholesterol; TG, triacylglycerol; AI, atherogenic index; p: significance of differences with the control group (mild COVID-19), corresponding genotype; Pcc, significance of differences with CC-genotype carriers in each group separately.

In the mild COVID-19 group, C-allele carriers rs1800790 had lower ET-1 and IL-6 levels than patients with TT-genotype by 34.63% and 36.60% (p = 0.034 and p = 0.048), respectively. In moderate-severe disease, TT-genotype carriers showed the highest ET-1 and PCT levels by 40.0-91.33%, suggesting stronger endothelial dysfunction and inflammatory activation associated with TT-genotype (p ≤ 0.049) (**Table 2**).

Metabolic pattern changes do not depend on rs2070744 (eNOS) gene polymorphism regardless the observed group (**Table 5**). All pro-atherogenic metabolic markers are higher in moderate-severe COVID-19 patients.

T-allele carriers of rs12329760 (TMPRSS2) showed higher TMPRSS2 blood value than CC-genotype carriers in the severe group by 13.82% (p = 0.048), whereas IL-6 levels were higher in CC-genotype carriers by 15.95% (p > 0.05) (**Table 3**). Overall, the data suggest genotype-dependent modulation of inflammatory and endothelial activation responses in COVID-19 severity.

In the mild COVID-19 group, T-allele carriers showed slightly lower HDL-C and TG values compared with CC-genotype carriers by 7.37% and 15.23% (p = 0.05 and p = 0.034), respectively. Overall, no major genotype-dependent differences were observed in severe disease, suggesting that metabolic disturbances are primarily driven by disease severity rather than rs12329760 variation (**Table 6**).

**Figure 1:**
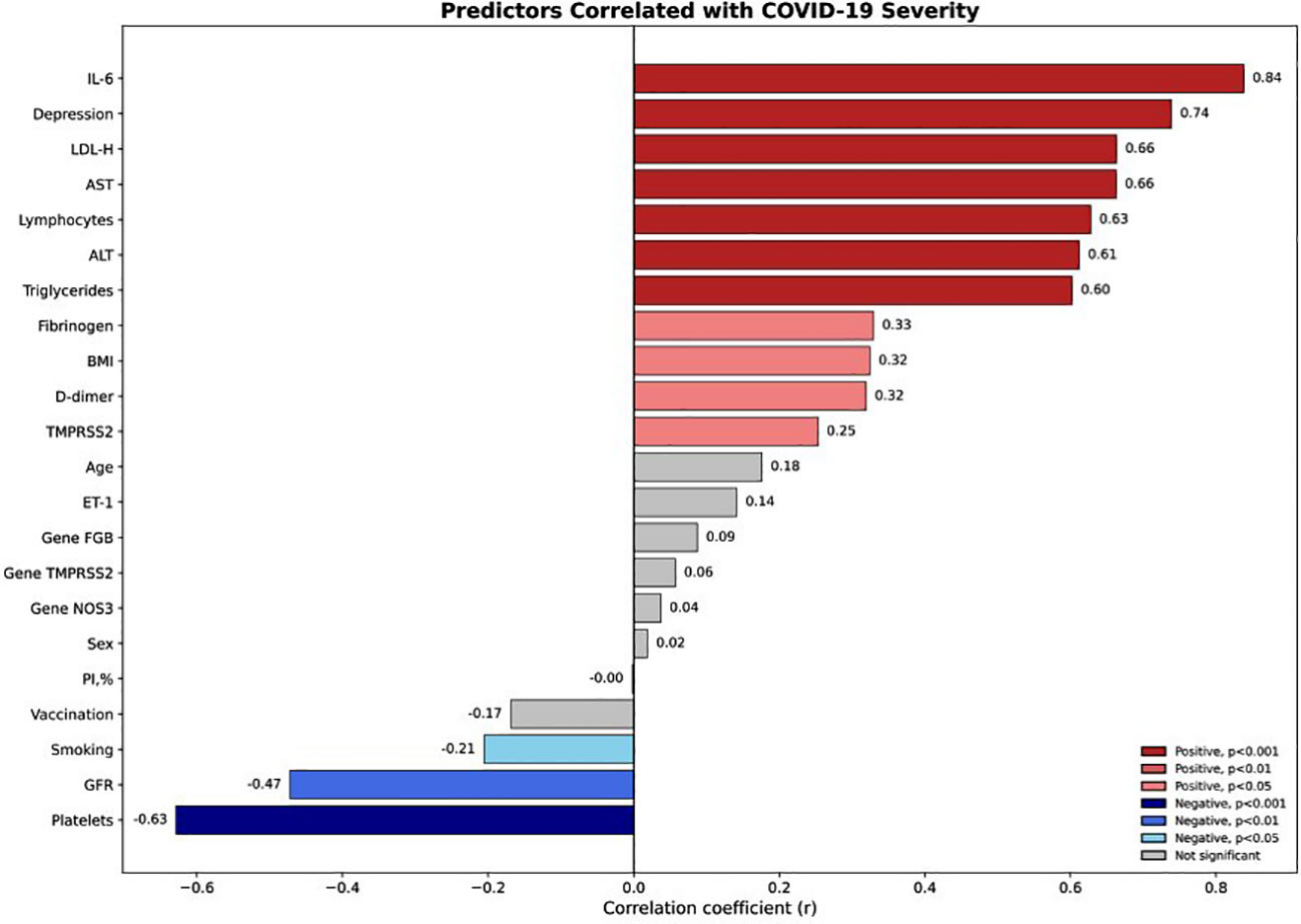
Correlation bar chart of clinical, biochemical, inflammatory, and molecular variables relative contributions to COVID-19 severity. Note. IL-6: interleukin-6; LDL-C: low density lipoprotein cholesterol; AST, ALT: Aspartate Aminotransferase, Alanine Aminotransferase; BMI: body mass index; TMPRSS2: transmembrane serine protease 2; ET-1: endothelin-1; PI: prothrombin index; GFR: glomerular filtration rate.

Correlation bar chart shows the strength and direction of associations with COVID-19 severity, illustrating the relative contribution of clinical, biochemical, inflammatory, and molecular variables to COVID-19 severity (**Figure 1**). Among all predictors, IL-6 demonstrated the strongest positive association with disease severity, followed by depression, LDL-C, Aspartate Aminotransferase (AST), lymphocyte count, Alanine Aminotransferase (ALT), and triglycerides. Moderate positive effects were also observed for fibrinogen, BMI, D-dimer, and TMPRSS2. In contrast, platelet count, glomerular filtration rate (GFR), and smoking showed inverse associations with severity. Overall, the figure highlights the dominant role of systemic inflammation, metabolic dysregulation, hepatic injury, and immune imbalance in severe COVID-19 progression.

Unlike the genetic association analyses, which compared patients with mild and moderate-to-severe disease, all machine-learning models were trained and evaluated using a three-class outcome (mild, moderate, and severe), allowing prediction across the full spectrum of COVID-19 severity.

**Figure 2:**
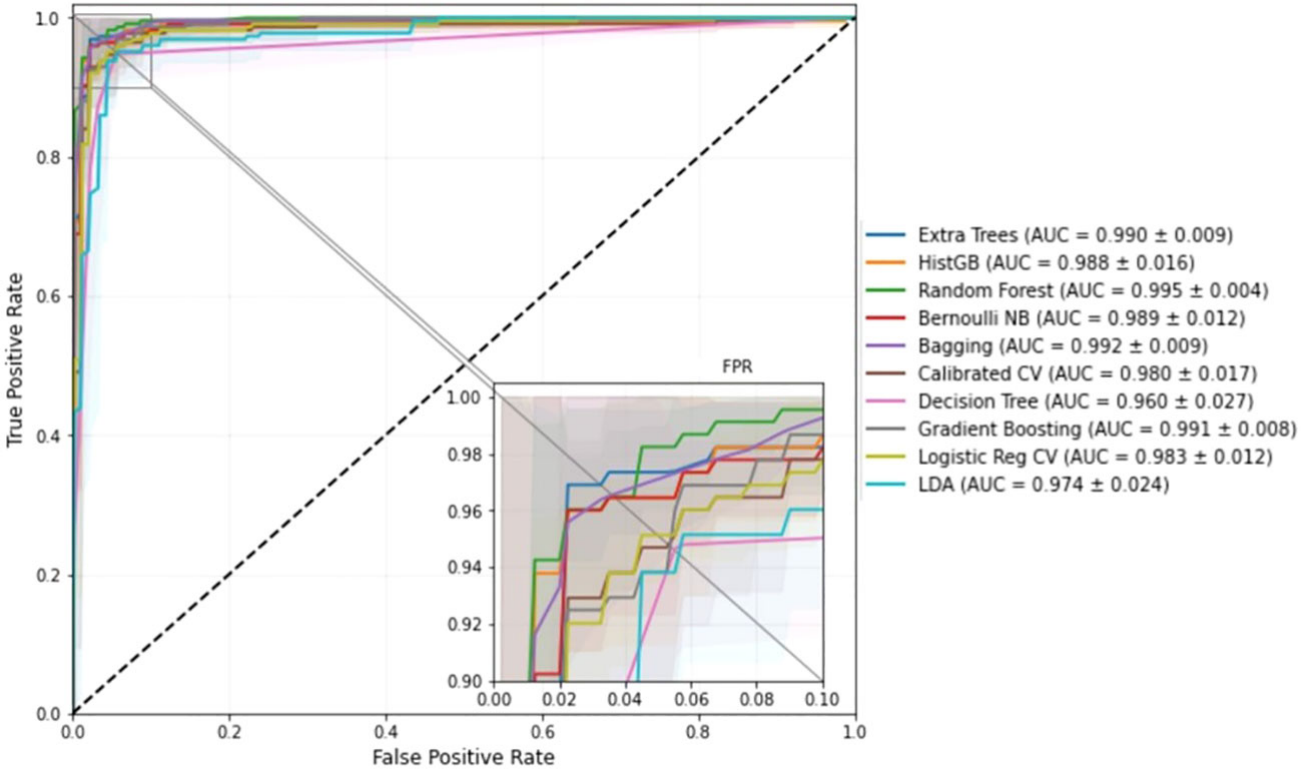
Weighted ROC curve for the top 10 classifiers in the multiclass classification task of COVID-19 severity, constructed using the One-vs-Rest (OvR) approach with 5-fold stratified cross-validation. Note. LDA: Linear Discriminant Analysis classifier.

In this study, the ROC curve was constructed for the top 10 classifiers identified in the previous analysis. Each model was evaluated using 5-fold stratified cross-validation to ensure the correct representation of all classes in both training and testing subsets. A weighted ROC metric was used to aggregate class-specific results into a single unified curve. The outcomes of ROC curve construction are presented in **Figure 2**. This figure displays the weighted ROC curve for each of the top 10 models, reflecting their aggregated performance in the multiclass classification task. All evaluated models demonstrated AUC-ROC values close to 1.0 (as noted in the previous section, the mean AUC-ROC for the top 10 models was 1.0), indicating a high ability of these classifiers to distinguish between the different severity categories of COVID-19. **Figure 2** illustrates that ensemble methods such as ExtraTreesClassifier (Accuracy: 0.974 ± 0.022) and RandomForestClassifier (Accuracy: 0.960 ± 0.035) exhibit ROC curves that closely approach the best classifier in our research (the upper-left corner of the plot). This confirms their superior performance in predicting COVID-19 severity. Simpler models, including BernoulliNB (Accuracy: 0.956 ± 0.037) and DecisionTreeClassifier (Accuracy: 0.938 ± 0.043), also demonstrated high classification quality, although their ROC curves show minor deviations from the best, likely due to sensitivity to noise or lower structural stability. The consistently high AUC-ROC values across all models indicate their strong capacity to effectively differentiate between severity classes of COVID-19.

The use of a weighted ROC curve provides a comprehensive evaluation of overall model performance while accounting for class imbalance in the dataset. However, the presence of near-ideal AUC-ROC values may also suggest potential overfitting or limited data variability within cross-validation folds, especially given the relatively small sample size (226 observations). Class imbalance particularly the limited representation of class 0 (30 samples) may influence performance, as models tend to achieve higher accuracy for the dominant class 2 (142 samples). The weighted ROC curves presented in **Figure 1** confirm the superior performance of the top 10 classifiers, particularly ensemble methods such as ExtraTreesClassifier, RandomForestClassifier, and HistGradientBoostingClassifier, in predicting COVID-19 severity. Although the AUC-ROC values (1.0) reflect the highest discriminatory capacity, external validation on an independent testing dataset is required to exclude potential overfitting. These findings form the basis for further hyperparameter optimization and subsequent development of a final model are promising for clinical application. External validation of the obtained results in larger multicentre cohorts is essential before clinical implementation.

To determine the optimal hyperparameters for the top 10 classifiers, we employed either grid search or random search optimization. For each model, a set of relevant hyperparameters was selected, including the number of trees in ensemble methods, tree depth, learning rate for gradient boosting algorithms, and regularization parameters for logistic regression, among others. Hyperparameter optimization was performed using 5-fold stratified cross-validation to ensure correct class representation in both training and testing subsets, given the imbalance of the target variable. Model performance was evaluated using Accuracy and AUC-ROC, consistent with previous stages of analysis, with a primary emphasis on maximizing AUC-ROC to ensure high discriminatory power. The optimal hyperparameters for each model were defined as the combination that achieved the highest mean AUC-ROC across cross-validation folds. To assess the importance of input features, we employed the permutation importance technique. Permutation importance is a model-agnostic method for evaluating feature relevance based on random permutation of feature values in the test dataset and quantifying the resulting degradation in model performance. Permutation importance was calculated within the cross-validation framework. For each validation fold, model performance was first calculated using the original validation data. The values of each feature were then randomly permuted, thereby disrupting its association with the outcome, and model performance was recalculated. Feature importance was defined as the decrease in the selected performance metric after permutation, with a larger reduction indicating a greater contribution of the feature to model performance. The procedure was repeated across cross-validation folds, and the resulting importance values were averaged to obtain more stable estimates.

This method is universal and applicable to any classifier, including both ensemble and linear models, because it does not depend on the model’s internal structure. Further-more, it captures nonlinear relationships and interactions among features, which is essential for our dataset, where correlation analysis revealed strong associations between Severity and variables such as IL-6 (0.838), Depression (0.739), and LDL-C (0.663).

Hyperparameter optimization improved the performance of the top 10 classifiers compared with their default configurations. Hyperparameter optimization was performed using GridSearchCV with stratified cross-validation. For each classifier, a predefined grid of relevant hyperparameters was evaluated, and the configuration yielding the best cross-validated performance was selected for final model training. The optimal hyperparameters for all models are reported in **Table 7**. Besides, the performance metrics of the optimized models, evaluated using 5-fold stratified cross-validation, precision, recall (sensitivity) and F1-score are presented in **Table 7**.

**Table 7:** Performance metrics of machine learning models for COVID-19 severity classification.

Models	Accuracy	AUC-ROC	Precision	Recall (sensitivity)	F1-score	Optimal hyperparameters
RandomForest	0.965	0.9998	0.955	0.944	0.949	max_depth: 20, min_samples_leaf: 2, min_samples_split: 2, n_estimators: 50
BernoulliNB	0.960	0.9879	0.871	0.890	0.880	alpha: 0.01, fit_prior: True
ExtraTrees	0.974	1.000	0.958	0.971	0.964	max_depth: 10, min_samples_split: 5, n_estimators: 200
CalibratedClassifierCV	0.943	0.9819	0.925	0.944	0.933	cv: 5, method:'sigmoid'
HistGradientBoosting	0.960	1.000	0.937	0.948	0.942	learning_rate: 0.2, max_depth: None, max_iter: 100
DecisionTree	0.960	0.9998	0.889	0.897	0.893	max_depth: 20, min_samples_leaf: 2, min_samples_split: 2
BaggingClassifier	0.969	1.000	0.941	0.955	0.946	max_features: 1.0, max_samples: 1.0, n_estimators: 50
LinearDiscriminantAnalysis	0.947	0.9778	0.912	0.926	0.918	shrinkage:'auto',solver: 'lsqr'
LogisticRegressionCV	0.960	0.9755	0.842	0.883	0.837	Cs: 100, max_iter: 1000, penalty:'l1',solver: 'liblinear'
GradientBoosting	0.938	1.000	0.911	0.924	0.918	learning_rate: 0.1, max_depth:3,n_estimators: 200

Hyperparameter optimization produced only modest changes in model performance compared with the baseline configurations. The largest improvements in accuracy were observed for BaggingClassifier, which increased from 0.951 ± 0.036 to 0.969 ± 0.033, LogisticRegressionCV, which increased from 0.934 ± 0.020 to 0.960 ± 0.026, and DecisionTreeClassifier, which increased from 0.938 ± 0.043 to 0.960 ± 0.022. RandomForestClassifier and LinearDiscriminantAnalysis also showed smaller improvements in accuracy, whereas the performance of ExtraTreesClassifier, HistGradientBoostingClassifier, GradientBoostingClassifier, and CalibratedClassifierCV remained largely unchanged. After optimization, ExtraTreesClassifier retained the highest accuracy of 0.974 ± 0.022, followed by BaggingClassifier at 0.969 ± 0.033 and RandomForestClassifier at 0.965 ± 0.033. Several models continued to show near-perfect AUC-ROC values, although these estimates should be interpreted cautiously given the internal cross-validation design and the relatively small sample size.

Ensemble-based algorithms demonstrated the strongest overall performance. The Extra Trees classifier achieved the highest precision (0.958), recall (0.971), and F1-score (0.964), followed closely by the Random Forest classifier (precision 0.955, recall 0.944, F1-score 0.949). These results indicate robust discrimination between mild and moderate–severe COVID-19 cases while maintaining a favourable balance between sensitivity and positive predictive value.

**Figure 3:**
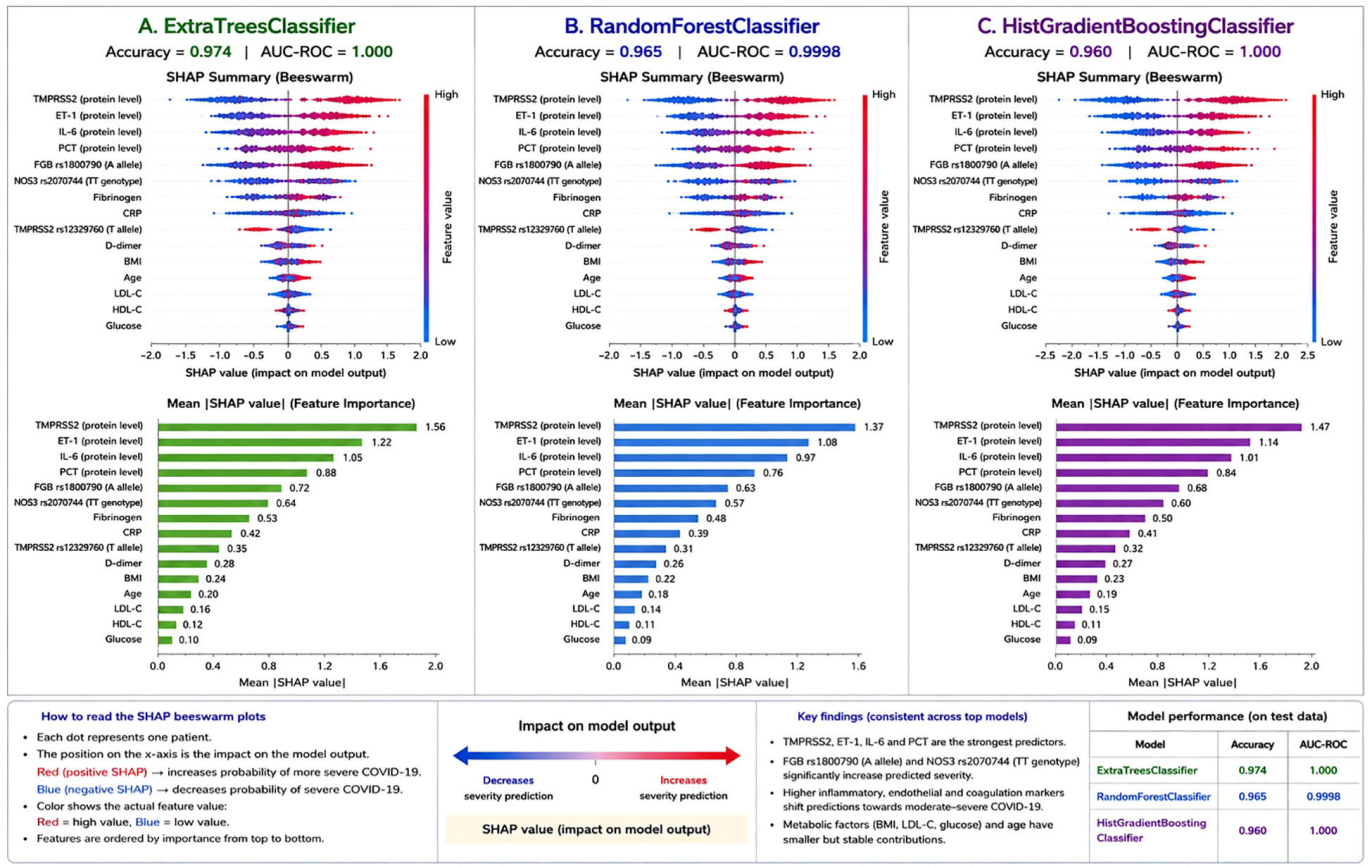
**(A-C)**. SHAP-based interpretability analysis of the three best-performing machine learning classifiers for COVID-19 severity prediction (moderate-severe course). Note. SHAP summary (beeswarm) plots and mean absolute SHAP value rankings are shown for ExtraTreesClassifier, RandomForestClassifier, and HistGradientBoostingClassifier. Each point represents an individual patient. Red points indicate higher feature values and blue points indicate lower feature values. Positive SHAP values increase the probability of classification as moderate-severe COVID-19, whereas negative SHAP values favor classification as mild disease. Transmembrane serine protease 2 (TMPRSS2) concentration, endothelin-1 (ET-1), interleukin-6 (IL-6), procalcitonin (PCT), FGB rs1800790, and NOS3 rs2070744 were consistently identified as the strongest contributors to model predictions across all classifiers.

To improve interpretability of the machine learning models and identify the variables contributing most strongly to COVID-19 severity prediction, SHAP analysis was performed for the three best-performing classifiers: ExtraTreesClassifier, RandomForestClassifier, and HistGradientBoostingClassifier (**Figure 3A-C**). All three models demonstrated highly consistent patterns of feature importance. TMPRSS2 protein concentration emerged as the strongest predictor of COVID-19 severity across all classifiers, exhibiting the highest mean absolute SHAP values. Endothelin-1 (ET-1), IL-6, and procalcitonin (PCT) ranked among the next most influential variables, indicating a substantial contribution of endothelial dysfunction and systemic inflammatory activation to disease severity prediction. Among genetic predictors, the FGB rs1800790 A allele and the NOS3 rs2070744 TT genotype consistently demonstrated significant contributions to model performance. The TMPRSS2 rs12329760 T allele showed a more moderate but reproducible effect across all models. Additional contributors included fibrinogen concentration, CRP, D-dimer, BMI, and age. The SHAP beeswarm plots further demonstrated that higher values of TMPRSS2, ET-1, IL-6, PCT, fibrinogen, and CRP shifted predictions toward the moderate–severe COVID-19 class, whereas lower values were associated with mild disease. Similarly, carriers of the FGB rs1800790 A allele and NOS3 rs2070744 TT-genotype exhibited a greater predicted probability of severe disease. The consistency of feature rankings across ExtraTreesClassifier (Accuracy = 0.974; AUC-ROC = 1.000), RandomForestClassifier (Accuracy = 0.965; AUC-ROC = 0.9998), and HistGradientBoostingClassifier (Accuracy = 0.960; AUC-ROC = 1.000) supports the robustness of the identified predictor set and suggests that disease severity is driven by converging mechanisms involving viral entry, endothelial injury, thromboinflammation, and host genetic susceptibility.

**Figure 4:**
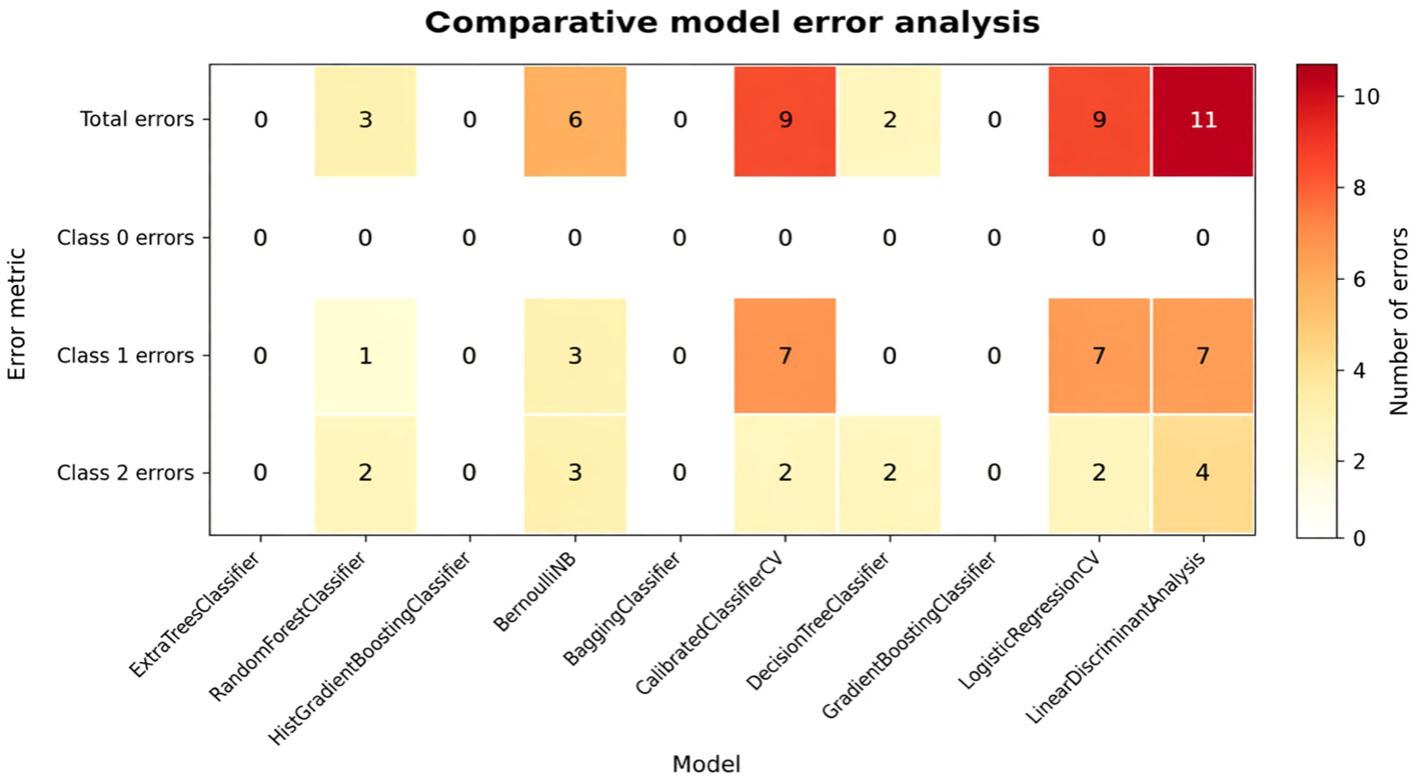
Performance of the classification models.

Performance of the classification models for the total number of errors depending on the disease severity for each ML classifier are presented in **Figure 4**. This analysis enabled the evaluation of performance for the ten best classification models used to predict COVID-19 severity and represent typical outcomes of error-analysis procedures. Error analysis included comparison of model predictions with true severity values, calculation of the total number of misclassifications, and assessment of error distribution across classes (0 class errors – mild COVID-19 course, 1 – moderate, 2 – severe).

Misclassifications occurrence can be explained by model complexity and their ability to capture non-linear relationships within the data. Linear models such as LogisticRegressionCV and LinearDiscriminantAnalysis assume linear separability and may therefore underperform in datasets characterized by complex, multidimensional interactions between clinical, biochemical, and genetic features. Similarly, CalibratedClassifierCV depends on an underlying base estimator and probability calibration, which may introduce additional variance and reduce classification precision in small or imbalanced datasets. In contrast, ensemble-based tree models are better suited to capture non-linear feature interactions and heterogeneous data structures, which likely contributed to their superior classification performance.

## 4 Discussion

COVID-19 severity is strongly associated with a pronounced pro-inflammatory and pro-endothelial activation profile. This profile is characterized by significant elevation of TMPRSS2, ET-1, IL-6, and PCT levels. It is also accompanied by marked metabolic dysregulation. Genetic variation in FGB rs1800790, eNOS rs2070744, and TMPRSS2 rs12329760 contributes to inter-individual differences in biomarker expression, with specific allelic variants modulating inflammatory intensity and endothelial dysfunction. In particular, the FGB rs1800790 A-allele and eNOS rs2070744 ТТ-genotypes are associated with a more pronounced inflammatory response and endothelial dysfunction, while rs12329760 TMPRSS2 variants show weaker but detectable modulatory effects with higher TMPRSS2 values in T-allele moderate-severe COVID-19 patients. No clear genotype-dependent differences in metabolic markers were observed for FGB rs1800790, NOS3 rs2070744, or TMPRSS2 rs12329760.

These findings support a more integrated understanding of COVID-19 pathogenesis, highlighting the complex interplay between endothelial dysfunction, pro- and anti-inflammatory responses, and genetically influenced immune regulation during SARS-CoV-2 infection. The observed biomarker profile (elevated TMPRSS2, IL-6, procalcitonin, and endothelin-1) indicates not only systemic inflammation but a dysregulated and potentially self-amplifying inflammatory state. In this context, the endothelium acts as an active effector of disease progression, contributing to microvascular injury, which may lead to systemic complications such as tissue hypoxia and life-threatening thrombosis (Bilous et al., 2020; Bilous et al., 2021; Chang, 2021; Stojanovic et al., 2026).

Genetic factors may significantly influence individual immune reactivity and susceptibility to severe outcomes following SARS-CoV-2 infection. In particular, the presence of the A-allele of FGB rs1800790 is correlated with a more intense inflammatory response, which can be associated with increased fibrinogen levels and, accordingly, an innate predisposition to hypercoagulability (Thachil, 2020; Kangro et al., 2022; de Maistre et al., 2023; Halabitska et al., 2025b). Similarly, it was found that the TT genotype variant for eNOS rs2070744 correlates with more pronounced endothelial dysfunction, which can be associated with insufficient vascular regulation and possibly with a decrease of nitric oxide bioavailability (Kamyshna et al., 2021; Montiel et al., 2022; Sydorchuk et al., 2023b; Teng et al., 2023).

Genetic variants of TMPRSS2 rs12329760 demonstrated a less striking modulating effect on the course of coronavirus disease, but we still found some significant correlations. High TMPRSS2 levels in T-allele patients with moderate and severe COVID-19 may indicate a mediating effect on the SARS-CoV-2 viral penetration severity (Lyubomirskaya et al., 2020; Vitello et al., 2022; Yaghoobi et al., 2023; Barros de Lima et al., 2025). In our opinion, despite the fact that the established interactions are not dominant, they should not be ignored in a comprehensive severe coronavirus disease risks assessment.

A major finding of the present study is the association between host genetic variability and the biological pathways underlying COVID-19 severity. Severe COVID-19 is increasingly recognized as a multifactorial disorder characterized by the interaction of viral entry mechanisms, endothelial dysfunction, thromboinflammation, and dysregulated immune responses. Our results suggest that polymorphisms in the FGB, NOS3, and TMPRSS2 genes may contribute to this pathological network. The association of the FGB rs1800790 polymorphism with disease severity is biologically plausible because fibrinogen is a central mediator of both coagulation and inflammation. Beyond its role as the precursor of fibrin, fibrinogen functions as an acute-phase reactant whose synthesis is markedly stimulated by pro-inflammatory cytokines, particularly IL-6 (Cronjé et al., 2017; de Maistre et al., 2023; Stojanovic et al., 2026). Increased fibrinogen levels promote platelet aggregation, fibrin formation, leukocyte recruitment, and endothelial activation, thereby contributing to the prothrombotic state observed in severe COVID-19 (Thachil, 2020; Chang, 2021; de Maistre et al., 2023). Carriage of the rs1800790 A allele has been associated with increased fibrinogen in our former study (Sokolenko et al., 2025), potentially predisposing individuals to enhanced coagulation activity and microvascular thrombosis (Cronjé et al., 2017). These mechanisms may explain the greater prevalence of severe disease among the risk allele carriers and support the concept of genetically mediated susceptibility to thromboinflammatory complications.

The NOS3 rs2070744 polymorphism may influence COVID-19 outcomes through its effects on endothelial nitric oxide synthase (eNOS) activity and nitric oxide (NO) bioavailability. Nitric oxide plays a critical role in maintaining vascular homeostasis by promoting vasodilation, inhibiting platelet aggregation, suppressing leukocyte adhesion, and limiting oxidative stress (Montiel et al., 2022; Sydorchuk et al., 2023b; Teng et al., 2023). Reduced NO production associated with the rs2070744 risk genotype may impair endothelial function and increase susceptibility to endothelial injury. Because endothelial dysfunction represents a hallmark of severe COVID-19, genetic factors affecting NO signalling may contribute directly to disease progression. This interpretation is further supported by the elevated endothelin-1 concentrations observed in our patients with severe disease, reflecting an imbalance between vasoprotective and vasoconstrictive endothelial pathways.

The TMPRSS2 rs12329760 polymorphism represents a distinct but complementary mechanism. TMPRSS2 encodes a serine protease required for SARS-CoV-2 spike protein priming and efficient viral entry into host cells. Functional variation within TMPRSS2 may influence viral infectivity, viral replication dynamics, and the subsequent intensity of the inflammatory response (Vitello et al., 2022; Yaghoobi et al., 2023; Barros de Lima et al., 2025; Sokolenko et al., 2025). Notably, circulating TMPRSS2 protein levels emerged as one of the strongest predictors of disease severity in our ML models, suggesting that activation of the TMPRSS2 pathway remains an important determinant of clinical outcome. The observed genetic associations therefore support a mechanistic link between host susceptibility to viral invasion and downstream inflammatory and endothelial responses.

Importantly, these pathways should not be viewed independently. Enhanced viral entry mediated through TMPRSS2 may amplify inflammatory activation, resulting in increased production of IL-6 and other cytokines. Elevated inflammatory signalling promotes fibrinogen synthesis and coagulation activation, while simultaneously inducing endothelial injury and dysfunction. Genetic predisposition affecting coagulation (FGB rs1800790) and endothelial regulation (NOS3 rs2070744) may therefore exacerbate these pathological processes. The convergence of these mechanisms is reflected in our biomarker profile characterized by elevated TMPRSS2, ET-1, IL-6, and procalcitonin levels in patients with more severe disease. Furthermore, SHAP-based model interpretation demonstrated that TMPRSS2, ET-1, IL-6, FGB rs1800790, and NOS3 rs2070744 consistently ranked among the most influential predictors across multiple ML algorithms. The concordance between biological plausibility, statistical associations, and explainable artificial intelligence analyses strengthens the hypothesis that host genetic variability contributes to COVID-19 severity through interconnected pathways involving viral entry, endothelial dysfunction, coagulation activation, and immune dysregulation.

We found any strong association between metabolic disorders and the studied genetic variants. This suggests that metabolic imbalance in COVID-19 is primarily driven by the systemic pro-inflammatory cytokine cascade and stress response rather than direct genetic determinants. However, the observed increase in pro-atherogenic indices in patients with severe COVID-19 indicates a potential long-term risk of cardiovascular complications, extending beyond the acute phase of infection (Cronjé et al., 2017; Bilous et al., 2020; Repchuk et al., 2021; Sydorchuk et al., 2023a).

Although biologically plausible candidate variants were selected based on their known involvement in coagulation, endothelial regulation, and SARS-CoV-2 host-cell interaction, the relatively modest sample size limits statistical power for genetic association analyses. Consequently, the observed associations should be considered exploratory and require confirmation in larger independent cohorts.

The findings of this study demonstrate a high effectiveness of ML approaches for classifying and predicting of COVID-19 severity based on a clinical and laboratory indicators’ comprehensive set. With the ten best classifiers performance, we identified important patterns related to model stability, accuracy, and generalizability. Our results are consistent with former research for predicting COVID-19 severity with ML approaches (Schwab et al., 2020; Wynants et al., 2020; Lages Dos Santos et al., 2025). Particular attention was given to ROC-AUC, Accuracy and SHAP analysis to identify the most relevant determinants of clinical disease progression (Assaf et al., 2020; Aktar et al., 2021; Chen et al., 2021; Ghaffar Nia et al., 2023; Sydorchuk et al., 2025; Fazilati et al., 2026; Sydorchuk et al., 2026). It should be noted that the imbalance among severity groups, with severe cases being the most prevalent, may have influenced model performance (Carrington et al., 2023; Al Hasanat et al., 2025; Debelie et al., 2026; Islam et al., 2026). However, the application of stratified cross-validation and weighted evaluation metrics was intended to mitigate potential bias. Despite this limitation, the ML models demonstrated the highest discriminatory performance, with AUC-ROC values approaching 1.0, indicating a near-perfect ability to distinguish between COVID-19 severity categories. These findings align with existing evidence reporting high predictive accuracy of ensemble-based ML methods in biomedical applications (Yang et al., 2022; Zafar et al., 2025; Xu, 2026).

We found that ensemble methods (ExtraTreesClassifier, RandomForestClassifier, and HistGradientBoostingClassifier) demonstrated the highest predictive performance, corroborating results of global meta-analyses in which these methods consistently showed optimal balance between sensitivity and specificity when applied to heterogeneous clinical and laboratory datasets (Yates et al., 2023; Al-Harbi et al., 2025; Khatami et al., 2026; Srivastava et al., 2026). Simpler models such as BernoulliNB and DecisionTreeClassifier also verified strong performance, although with reduced stability, an observation that echoes previous reports highlighting their sensitivity to multicollinearity and data noise (Mahajan et al., 2023; Halabitska et al., 2024; Muhammad et al., 2024; Halabitska et al., 2025a). Hyperparameter optimization improved the predictive accuracy of several models, including LogisticRegressionCV and BaggingClassifier, underscoring the importance of fine-tuning in applied clinical machine-learning tasks.

In the internal error analysis, several ensemble models produced no misclassifications in the evaluated prediction output. However, this finding should not be interpreted as evidence of exceptional generalizability because no independent external validation was performed. In contrast, linear models such as LinearDiscriminantAnalysis and LogisticRegressionCV, as well as CalibratedClassifierCV generated the highest number of errors than ensemble-based approaches, due to their assumptions regarding linear decision boundaries and limited ability to capture complex non-linear interactions among genetic, biochemical, and clinical variables (Ushasree et al., 2024; Bangash et al., 2025; Mahmood et al., 2025). Although several models achieved exceptionally high performance metrics, the idealized AUC-ROC values warrant cautious interpretation. Given the relatively small sample size, the risk of overfitting should not be overlooked, even when stratified cross-validation is employed (Montesinos López et al., 2022; Al Duhayyim et al., 2023; Li et al., 2025; Imani et al., 2026). Therefore, in our view, further external validation on independent datasets remains a critical next step for confirming the clinical applicability of these models.

Overall, the ensemble-based models in our study reaffirm their leading role in high-precision prediction of clinical complications associated with COVID-19 (Vabalas et al., 2019; Leader Lawanga et al., 2023; Rane et al., 2024).

One of the major strengths of the present study is the integration of explainable AI approaches with genetic and biomarker analyses (Bajwa et al., 2021; Kaur et al., 2021; Jamarani et al., 2024; Fahim et al., 2025; Gawande et al., 2025; Rabbani et al., 2025). While ensemble ML algorithms demonstrated the highest discriminatory performance, SHAP analysis provided important biological insight into the mechanisms underlying model predictions. The identification of circulating TMPRSS2, Endothelin-1 and inflammatory biomarkers, particularly IL-6 and procalcitonin levels, as the most influential predictors across all models are biologically plausible, and are among the strongest contributors to model performance. These findings are consistent with previous reports linking cytokine dysregulation and hyperinflammatory responses to disease severity, as well as vasoconstriction, oxidative stress, leukocyte recruitment, platelet activation, microvascular thrombosis, organ dysfunction, and mortality (Herold et al., 2020; Lippi and Plebani, 2020; Tang et al., 2020; Powell et al., 2021; Rahnavard et al., 2022; Choudhri et al., 2023; Sokolenko et al., 2024; Sokolenko et al., 2025; Stec-Martyna et al., 2025; Li et al., 2026). Moreover, TMPRSS2 is a key host protease facilitating SARS-CoV-2 spike protein priming and viral entry into target cells. Elevated TMPRSS2 expression may therefore reflect enhanced susceptibility to viral invasion and greater tissue damage, ultimately contributing to more severe clinical manifestations (Vitello et al., 2022; Yaghoobi et al., 2023; Barros de Lima et al., 2025). Importantly, SHAP analysis identified FGB rs1800790 and NOS3 rs2070744 as the most influential genetic predictors. The FGB rs1800790 A allele has been associated with increased fibrinogen expression and enhanced thrombotic potential (Cronjé et al., 2017; Sokolenko et al., 2025). Elevated fibrinogen concentrations promote fibrin formation, platelet aggregation, endothelial activation, and immunothrombosis, all of which are hallmarks of severe COVID-19 (Thachil, 2020; Chang, 2021; Kangro et al., 2022; Montiel et al., 2022; de Maistre et al., 2023; Stojanovic et al., 2026). Similarly, the NOS3 rs2070744 polymorphism (particularly TT-genotype) influences endothelial nitric oxide synthase activity and nitric oxide bioavailability. Reduced nitric oxide production may impair vasodilation, increase oxidative stress, and exacerbate endothelial dysfunction. The prominent contribution of FGB rs1800790 and NOS3 rs2070744 variants supports the hypothesis that inherited differences in coagulation pathways and genetic regulation of endothelial homeostasis may partly explain individual variability in disease severity and represents key determinants of COVID-19 outcomes. The broadly consistent feature-importance rankings across Extra Trees, Random Forest, and HistGradientBoosting classifiers support the internal stability of the identified predictor pattern. Nevertheless, all algorithms were evaluated using the same dataset, and this consistency does not replace external validation. Although the predictive performance of the models was exceptionally high, the absence of external validation warrants caution. Nevertheless, the stability of SHAP-derived feature rankings across multiple independent algorithms suggests that the identified predictors represent biologically meaningful signals rather than model-specific artefacts. Future multicentre studies with larger populations and external validation cohorts will be necessary to confirm these observations and assess their clinical utility.

The candidate-gene approach adopted in the present study was based on biological plausibility and previous evidence supporting the involvement of FGB, NOS3, and TMPRSS2 in COVID-19 pathophysiology (Sokolenko et al., 2024; Sokolenko et al., 2025; Sydorchuk et al., 2025). Nevertheless, the relatively modest sample size available for genotyping limits the statistical power to detect small genetic effects, particularly for less frequent genotypes. Although the observed associations were biologically consistent and aligned with our previous findings, they should be interpreted cautiously and regarded as hypothesis-generating until confirmed in larger, independent cohorts and genome-wide investigations.

Several limitations should be acknowledged. First, the sample size was modest relative to the number of candidate predictors, increasing the risk of overfitting and limiting the precision of the genetic association estimates. Second, model performance was assessed using internal stratified cross-validation only, and no independent hold-out or external validation cohort was available. Therefore, the reported performance estimates may be optimistic.

Third, the severity classes were imbalanced, and no explicit resampling or class-weighting strategy was applied. Weighted performance metrics may consequently have been influenced by the predominance of the largest class. The original analytical output did not include balanced accuracy or class-specific specificity, limiting the assessment of model performance across all severity categories.

Fourth, several clinical variables were closely related to the criteria used to define disease severity. The present results should therefore be interpreted primarily as classification of observed COVID-19 severity rather than prospective prediction of future clinical deterioration. Finally, genotyping was available only for a subset of participants, limiting the statistical power and generalizability of the integrated genetic findings. All genetic and machine-learning findings should be considered exploratory until confirmed in larger, independent multicentre cohorts.

## 5 Conclusions

COVID-19 severity is strongly associated with a pronounced pro-inflammatory and pro-endothelial activation profile, characterized by significantly elevated TMPRSS2, ET-1, IL-6, and PCT levels, together with marked metabolic dysregulation. Genetic variation in FGB rs1800790, eNOS rs2070744, and TMPRSS2 rs12329760 contributes to inter-individual differences in biomarker expression, with specific allelic variants modulating inflammatory intensity and endothelial dysfunction. In particular, the FGB rs1800790 A-allele and eNOS rs2070744 ТТ-genotypes are associated with a more pronounced inflammatory response and endothelial dysfunction, while rs12329760 TMPRSS2 variants show weaker but detectable modulatory effects with higher TMPRSS2 values in T-allele moderate-severe COVID-19 patients. Metabolic pattern changes do not depend on rs1800790 (FGB), rs2070744 (eNOS), and rs12329760 (TMPRSS2) genes polymorphism. All pro-atherogenic metabolic markers are higher in moderate-severe COVID-19 patients. Overall, the findings suggest a combined genetic-biomarker signature that reflects disease severity and may support individualized risk stratification in COVID-19.

In the exploratory machine-learning analysis, ensemble classifiers, particularly ExtraTreesClassifier and RandomForestClassifier, showed the highest internal cross-validated performance for classifying COVID-19 severity. SHAP analysis identified TMPRSS2, ET-1, IL-6, PCT, fibrinogen, CRP, and selected genetic variables as important contributors to model predictions.

Nevertheless, the near-perfect weighted AUC-ROC values should not be interpreted as evidence of established clinical utility. The relatively small and imbalanced dataset, the number of candidate predictors, and the absence of independent external validation create a substantial risk of optimistic performance estimation. Therefore, the present results should be regarded as exploratory and hypothesis-generating. Validation in larger, independent multicentre cohorts is required before these models can be used for prospective risk prediction or clinical decision-making.

The SHAP analysis demonstrated that higher values of TMPRSS2, ET-1, IL-6, PCT, fibrinogen, and CRP shifted predictions toward the moderate–severe COVID-19 class, whereas lower values were associated with mild disease. Similarly, carriers of the FGB rs1800790 A allele and NOS3 rs2070744 TT-genotype exhibited a greater predicted probability of severe disease. The consistency of feature importance rankings across Extra Trees, Random Forest, and HistGradientBoosting classifiers strengthens the biological credibility of these findings.

External validation of the obtained results in larger multicentre cohorts is essential before clinical implementation.

## Data Availability

The raw data supporting the conclusions of this article will be made available by the authors, without undue reservation.

## References

[B1] Abbas Q. Jeong W. Lee S. W. (2025). Explainable AI in clinical decision support systems: a meta-analysis of methods, applications, and usability challenges. Healthcare 13, 2154. doi: 10.3390/healthcare13172154 40941506 PMC12427955

[B2] Aktar S. Ahamad M. M. Rashed-Al-Mahfuz M. Azad A. Uddin S. Kamal A. . (2021). Machine learning approach to predicting COVID-19 disease severity based on clinical blood test data: statistical analysis and model development. JMIR Med. Inf. 9, e25884. doi: 10.2196/25884 33779565 PMC8045777

[B3] Al Duhayyim M. Abbas S. Hejaili A. Kryvinska N. Almadhor A. Mohammad U. (2023). An ensemble machine learning technique for stroke prognosis. Comput. Syst. Sci. Eng. 47, 413–429. doi: 10.32604/csse.2023.037127

[B4] Al-Harbi F. A. AlKuwaiti M. A. Alharbi M. A. Alessa A. A. Alhassan A. A. Aleidan E. A. . (2025). Diagnostic accuracy of artificial intelligence in predicting anti-VEGF treatment response in diabetic macular edema: a systematic review and meta-analysis. J. Clin. Med. 14, 8177. doi: 10.3390/jcm14228177 41303218 PMC12653470

[B5] Al Hasanat H. A. Hassanat A. B. Alharasees O. Tarawneh A. S. Altarawneh G. A. Alhasanat L. A. . (2025). Interpretable machine learning for imbalanced pedestrian injury severity prediction in urban Jordan. J. King Saud Univ. Eng. Sci. 37, 39. doi: 10.1007/s44444-025-00053-3 30311153

[B6] Assaf D. Gutman Y. Neuman Y. Segal G. Amit S. Gefen-Halevi S. . (2020). Utilization of machine-learning models to accurately predict the risk for critical COVID-19. Internal Emergency Med. 15, 1435–1443. doi: 10.1007/s11739-020-02475-0 32812204 PMC7433773

[B7] Bajwa J. Munir U. Nori A. Williams B. (2021). Artificial intelligence in healthcare: transforming the practice of medicine. Future Healthcare J. 8, e188-e94. doi: 10.7861/fhj.2021-0095 34286183 PMC8285156

[B8] Bangash A. H. Bercu M. M. Byrne R. W. Pavuluri S. Salehi A. (2025). Application of machine learning approaches to predict seizure-onset zones in patients with drug-resistant epilepsy: a systematic review. Front. Neurol. 16. doi: 10.3389/fneur.2025.1687144 41404463 PMC12702746

[B9] Barros de Lima G. Nencioni E. Thimoteo F. Perea C. Pinto R. F. A. Sasaki S. D. (2025). TMPRSS2 as a key player in viral pathogenesis: influenza and coronaviruses. Biomolecules 15, 75. doi: 10.3390/biom15010075 39858469 PMC11764435

[B10] Bilous I. I. Korda M. M. Krynytska I. Y. Kamyshnyi A. M. (2020). Nerve impulse transmission pathway-focused genes expression analysis in patients with primary hypothyroidism and autoimmune thyroiditis. Endocrine Regulations 54, 109–118. doi: 10.2478/enr-2020-0013 32597152

[B11] Bilous I. I. Pavlovych L. L. Kamyshnyi A. M. (2021). Primary hypothyroidism and autoimmune thyroiditis alter the transcriptional activity of genes regulating neurogenesis in the blood of patients. Endocrine Regulations 55, 5–15. doi: 10.2478/enr-2021-0002 33600668

[B12] Bilous I. Pavlovych L. Krynytska I. Marushchak M. Kamyshnyi O. (2020). Apoptosis and cell cycle pathway-focused genes expression analysis in patients with different forms of thyroid pathology. Open Access Macedonian J. Med. Sci. 8, 784–792. doi: 10.3889/oamjms.2020.4760

[B13] Bosello S. De Luca G. Berardi G. Canestrari G. de Waure C. Gabrielli F. A. . (2019). Cardiac troponin T and NT-proBNP as diagnostic and prognostic biomarkers of primary cardiac involvement and disease severity in systemic sclerosis: a prospective study. Eur. J. Intern. Med. 60, 46–53. doi: 10.1016/j.ejim.2018.10.013 30366614

[B14] Buttia C. Llanaj E. Raeisi-Dehkordi H. Kastrati L. Amiri M. Meçani R. . (2023). Prognostic models in COVID-19 infection that predict severity: a systematic review. Eur. J. Epidemiol. 38, 355–372. doi: 10.1007/s10654-023-00973-x 36840867 PMC9958330

[B15] Carrington A. M. Manuel D. G. Fieguth P. W. Ramsay T. Osmani V. Wernly B. . (2023). Deep ROC analysis and AUC as balanced average accuracy, for improved classifier selection, audit and explanation. IEEE Trans. Pattern Anal. Mach. Intell. 45, 329–341. doi: 10.1109/tpami.2022.3145392 35077357

[B16] Prevention Centers for Disease C (2023). CDC 24/7: saving lives, protecting people. Prevention actions to use at all COVID-19 community levels.

[B17] Chang J. C. (2021). COVID-19 sepsis: pathogenesis and endothelial molecular mechanisms based on "two-path unifying theory" of hemostasis and endotheliopathy-associated vascular microthrombotic disease, and proposed therapeutic approach with antimicrothrombotic therapy. Vasc. Health Risk Manage. 17, 273–298. doi: 10.2147/vhrm.s299357 34103921 PMC8179800

[B18] Chen Y. Ouyang L. Bao F. S. Li Q. Han L. Zhang H. . (2021). A multimodality machine learning approach to differentiate severe and nonsevere COVID-19: model development and validation. J. Med. Internet Res. 23, e23948. doi: 10.2196/23948 33714935 PMC8030658

[B19] Choudhri Y. Maslove D. M. Rauh M. J. (2023). COVID-19 and the genetics of inflammation. Crit. Care Med. 51, 817–825. doi: 10.1097/ccm.0000000000005843 36939259 PMC10187540

[B20] Cronjé H. T. Nienaber-Rousseau C. Zandberg L. de Lange Z. Green F. R. Pieters M. (2017). Fibrinogen and clot-related phenotypes determined by fibrinogen polymorphisms: independent and IL-6-interactive associations. PloS One 12, e0187712. 29099861 10.1371/journal.pone.0187712 PMC5669433

[B21] Debelie A. Bagui S. S. Mink D. Bagui S. C. (2026). Class-specific GAN augmentation for imbalanced intrusion detection: a comparative study using the UWF-ZeekData22 dataset. Future Internet 18, 200. doi: 10.3390/fi18040200 30654563

[B22] de Maistre E. Savard P. Guinot P. G. (2023). COVID-19 and the concept of thrombo-inflammation: review of the relationship between immune response, endothelium and coagulation. J. Clin. Med. 12. doi: 10.3390/jcm12237245 38068297 PMC10706970

[B23] De Simone B. Abu-Zidan F. M. Chouillard E. Di Saverio S. Sartelli M. Podda M. . (2022). The ChoCO-W prospective observational global study: does COVID-19 increase gangrenous cholecystitis? World J. Emergency Surg. WJES 17, 61. doi: 10.1186/s13017-022-00466-4 36527038 PMC9755784

[B24] De Simone B. Abu-Zidan F. M. Saeidi S. Deeken G. Biffl W. L. Moore E. E. . (2024). Knowledge, attitudes and practices of using indocyanine green (ICG) fluorescence in emergency surgery: an international web-based survey in the ARtificial Intelligence in Emergency and trauma Surgery (ARIES)-WSES project. Updates Surg. 76, 1969–1981. doi: 10.1007/s13304-024-01853-z 38801604

[B25] Eini P. Rezayee M. Kassulke M. Tremblay J. (2026). Efficacy and comparative performance of machine learning models for stroke risk prediction in hypertensive patients: a systematic review and meta-analysis. Int. J. Cardiol. Cardiovasc. Risk Prev. 28, 200564. doi: 10.1016/j.ijcrp.2025.200564 41550126 PMC12811527

[B26] El-Kassas M. Alboraie M. Elbadry M. El Sheemy R. Abdellah M. Afify S. . (2023). Non-pulmonary involvement in COVID-19: a systemic disease rather than a pure respiratory infection. World J. Clin. cases 11, 493–505. doi: 10.12998/wjcc.v11.i3.493 36793640 PMC9923857

[B27] Esmaeilzadeh A. Elahi R. Siahmansouri A. Maleki A. J. Moradi A. (2022). Endocrine and metabolic complications of COVID-19: lessons learned and future prospects. J. Mol. Endocrinol. 69, R125–R150. doi: 10.1530/jme-22-0036 35900847

[B28] Eze C. Igwama G. Emeihe E. (2024). AI-driven health data analytics for early detection of infectious diseases: a conceptual exploration of U.S. public health strategies. Compr. Res. Rev. Sci. Technol. 2. doi: 10.57219/crrst.2024.2.2.0038

[B29] Fahim Y. A. Hasani I. W. Kabba S. Ragab W. M. (2025). Artificial intelligence in healthcare and medicine: clinical applications, therapeutic advances, and future perspectives. Eur. J. Med. Res. 30, 848. doi: 10.1186/s40001-025-03196-w 40988064 PMC12455834

[B30] Fazilati F. Rajabi M. Z. Alihosseini N. Farsani M. E. Sandid S. H. Zamani S. . (2026). TRIAGE: trustworthy reporting and assessment for clinical gain and effectiveness of AI models. Diagnostics (Basel Switzerland) 16. doi: 10.3390/diagnostics16050666 41827942 PMC12984829

[B31] Fu Y. Zhong W. Liu T. Li J. Xiao K. Ma X. . (2022). Early prediction model for critical illness of hospitalized COVID-19 patients based on machine learning techniques. Front. Public Health 10, 880999. doi: 10.3389/fpubh.2022.880999 35677769 PMC9168534

[B32] Gao L. Jiang D. Wen X. S. Cheng X. C. Sun M. He B. . (2020). Prognostic value of NT-proBNP in patients with severe COVID-19. Respir. Res. 21, 83. doi: 10.1186/s12931-020-01352-w 32293449 PMC7156898

[B33] Gawande M. S. Zade N. Kumar P. Gundewar S. Weerarathna I. N. Verma P. (2025). The role of artificial intelligence in pandemic responses: from epidemiological modeling to vaccine development. Mol. Biomed. 6, 1. doi: 10.1186/s43556-024-00238-3 39747786 PMC11695538

[B34] Ghaffar Nia N. Kaplanoglu E. Nasab A. (2023). Evaluation of artificial intelligence techniques in disease diagnosis and prediction. Discover Artif. Intell. 3, 5. doi: 10.1007/s44163-023-00049-5 40478140 PMC9885935

[B35] Halabitska I. Petakh P. Buchynskyi M. Kamyshna I. Lushchak O. Kamyshnyi O. (2025a). War, diet, and PTSD in Ukrainian youth. Sci. Rep. 16, 1422. doi: 10.1038/s41598-025-31138-3 41345238 PMC12796481

[B36] Halabitska I. Petakh P. Kamyshnyi O. (2025b). Metformin as a disease-modifying therapy in osteoarthritis: bridging metabolism and joint health. Front. Pharmacol. 16, 1567544. doi: 10.3389/fphar.2025.1567544 40176893 PMC11962732

[B37] Halabitska I. Petakh P. Oksenych V. Kamyshnyi O. (2024). Predictive analysis of osteoarthritis and chronic pancreatitis comorbidity: complications and risk factors. Front. Endocrinol. 15, 1492741. doi: 10.20944/preprints202407.1125.v1 PMC11576156 39568811

[B38] He X. Ding Q. (2024). D-dimer-to-platelet count ratio as a novel indicator for predicting prognosis in HBV-related decompensated cirrhosis. Heliyon 10, e26585. doi: 10.1016/j.heliyon.2024.e26585 38434313 PMC10907634

[B39] Herold T. Jurinovic V. Arnreich C. Lipworth B. J. Hellmuth J. C. von Bergwelt-Baildon M. . (2020). Elevated levels of IL-6 and CRP predict the need for mechanical ventilation in COVID-19. J. Allergy Clin. Immunol. 146, 128–136.e4. doi: 10.1016/j.jaci.2020.05.008 32425269 PMC7233239

[B40] Imani M. Joudaki M. Bagheri A. Arabnia H. R. (2026). Why ROC-AUC is misleading for highly imbalanced data: in-depth evaluation of MCC, F2-score, H-measure, and AUC-based metrics across diverse classifiers. Technologies 14, 54. doi: 10.20944/preprints202510.0958.v2

[B41] Islam S. Haque R. Khan M. A. Mohiuddin A. B. Hossain Siddiqui M. I. Limon Z. H. . (2026). Ensemble transformer with post-hoc explanations for depression emotion and severity detection. iScience 29, 114605. doi: 10.1016/j.isci.2025.114605 41630912 PMC12860732

[B42] Jabczyk M. Nowak J. Hudzik B. Zubelewicz-Szkodzińska B. (2021). Microbiota and its impact on the immune system in COVID-19-a narrative review. J. Clin. Med. 10. doi: 10.3390/jcm10194537 34640553 PMC8509181

[B43] Jamarani A. Haddadi S. Sarvizadeh R. Haghi Kashani M. Akbari M. Moradi S. (2024). Big data and predictive analytics: a systematic review of applications. Artif. Intell. Rev. 57, 176. doi: 10.1007/s10462-024-10811-5 30311153

[B44] Kamyshna I. I. Pavlovych L. B. Maslyanko V. A. Kamyshnyi A. M. (2021). Analysis of the transcriptional activity of genes of neuropeptides and their receptors in the blood of patients with thyroid pathology. J. Med. Life. 14, 243–249. doi: 10.25122/jml-2020-0183 34104248 PMC8169137

[B45] Kangro K. Wolberg A. S. Flick M. J. (2022). Fibrinogen, fibrin, and fibrin degradation products in COVID-19. Curr. Drug Targets 23, 1593–1602. doi: 10.2174/1389450123666220826162900 36029073 PMC10316333

[B46] Kasundra P. R. Ghada V. J. (2026). A systematic review of machine learning techniques for cardiovascular disease prediction with performance analysis and future perspectives. Cureus J. Comput. Sci. 3, es44389-026-00060-8. doi: 10.7759/s44389-026-00060-8 32778053

[B47] Kaur I. Behl T. Aleya L. Rahman H. Kumar A. Arora S. . (2021). Artificial intelligence as a fundamental tool in management of infectious diseases and its current implementation in COVID-19 pandemic. Environ. Sci. pollut. Res. Int. 28, 40515–40532. doi: 10.1007/s11356-021-13823-8 34036497 PMC8148397

[B48] Khan S. Rasool S. T. Ahmed S. I. (2021). Role of cardiac biomarkers in COVID-19: what recent investigations tell us? Curr. Probl. Cardiol. 46, 100842. doi: 10.1016/j.cpcardiol.2021.100842 33994028 PMC7977033

[B49] Khatami S. Faghihi M. Irajian P. Jafari-Nakhjavanlou A. Khatami H. Khatami R. . (2026). Comparing machine learning models for predicting mortality after myocardial infarction: a systematic review and meta-analysis. Arch. Acad. Emergency Med. 14, e2. 10.22037/aaem.v14i1.2783 PMC12883175 41664678

[B50] Klaudel T. Pelczarski M. Zaborska M. Sadowski J. Ostrowska S. A. Drzymała A. . (2025). The impact of diabetes and obesity on the severity and mortality of SARS-CoV-2 infection. J. Diabetes Metab. Disord. 24, 195. doi: 10.1007/s40200-025-01706-5 40904859 PMC12401857

[B51] Kolesnyk P. O. Paliy I. H. Sydorchuk L. P. Hoda Z. P. Ivanchenko N. O. Lych O. S. . (2024). The role of nutritional support with probiotics in outpatients with symptomatic acute respiratory tract infections: a multicenter, randomized, double-blind, placebo-controlled dietary study. BMC Nutr. 10, 4. doi: 10.1186/s40795-023-00816-8 38178223 PMC10768308

[B52] Koteluk O. Wartecki A. Mazurek S. Kołodziejczak I. Mackiewicz A. (2021). How do machines learn? Artificial intelligence as a new era in medicine. J. Personalized Med. 11. doi: 10.3390/jpm11010032 33430240 PMC7825660

[B53] Lages Dos Santos A. Oliveira M. C. L. Colosimo E. A. Mak R. H. Pinhati C. C. Gallante S. C. . (2025). Comparative performance of twelve machine learning models in predicting COVID-19 mortality risk in children: a population-based retrospective cohort study in Brazil. PeerJ Comput. Sci. 11, e2916. doi: 10.7717/peerj-cs.2916 40567691 PMC12192853

[B54] Lai C. C. Shih T. P. Ko W. C. Tang H. J. Hsueh P. R. (2020). Severe acute respiratory syndrome coronavirus 2 (SARS-CoV-2) and coronavirus disease-2019 (COVID-19): the epidemic and the challenges. Int. J. Antimicrob. Agents 55, 105924. doi: 10.1016/j.ijantimicag.2020.105924 32081636 PMC7127800

[B55] Leader Lawanga O. Jean-Christophe Mulangu S. Placide Mbala K. Olivier Tshiani M. Jean-Michel Nsengi N. Jean-jacque Muyembe T. . (2023). Comparison of the performance of linear discriminant analysis and binary logistic regression applied to risk factors for mortality in Ebola virus disease patients. J. Electronics Electromedical Engineering Med. Inf. 5. doi: 10.35882/jeeemi.v5i3.303

[B56] Li J. Du X. Zhang R. Li X. Xu J. Song X. . (2025). Machine learning models for predicting short-term progression in patients with stage 4 chronic kidney disease: a multi-center validation study. Sci. Rep. 15, 39285. doi: 10.1038/s41598-025-23037-4 41214104 PMC12603016

[B57] Li X. Lv J. Guo H. Li H. Zhao Q. Qi H. . (2026). Development and internal validation of an early warning predictive model for critically ill patients in the emergency department utilizing easily obtainable clinical indicators. Risk Manage. Healthcare Policy 19, 566920. doi: 10.2147/rmhp.s566920 41783643 PMC12954202

[B58] Li M. Sharma K. Chon J.-E. Yehia N. A. Retnakaran R. Harris S. B. . (2026). The impact of COVID-19 illness on metabolic phenotypes underlying type 2 diabetes mellitus: a systematic review. Diabetes Res. Clin. Pract. 235, 113163. doi: 10.1016/j.diabres.2026.113163 41763558

[B59] Li J. Zhao Q. Gao H. Wang H. Guo C. Feng X. (2026). Early biomarkers for predicting sepsis-induced shock: insights from inflammatory pathways and immune response. Front. Pharmacol. 17, 1751781. doi: 10.3389/fphar.2026.1751781 41868133 PMC12999895

[B60] Li X. Zhou X. Ding S. Chen L. Feng K. Li H. . (2022). Identification of transcriptome biomarkers for severe COVID-19 with machine learning methods. Biomolecules 12. doi: 10.3390/biom12121735 36551164 PMC9775121

[B61] Lippi G. Plebani M. (2020). Procalcitonin in patients with severe coronavirus disease 2019 (COVID-19): a meta-analysis. Clinica Chimica Acta; Int. J. Clin. Chem. 505, 190–191. doi: 10.1016/j.cca.2020.03.004 32145275 PMC7094472

[B62] Liu T. Krentz A. J. Huo Z. Ćurčin V. (2025). Opportunities and challenges of cardiovascular disease risk prediction for primary prevention using machine learning and electronic health records: a systematic review. Rev. Cardiovasc. Med. 26, 37443. doi: 10.31083/rcm37443 40351688 PMC12059770

[B63] Lyubomirskaya E. S. Kamyshnyi A. M. Krut Y. Y. Smiianov V. A. Fedoniuk L. Y. Romanyuk L. B. . (2020). SNPs and transcriptional activity of genes of innate and adaptive immunity at the maternal-fetal interface in woman with preterm labour, associated with preterm premature rupture of membranes. Wiadomosci Lekarskie (Warsaw Poland 1960) 73, 25–30. doi: 10.36740/wlek202001104 32124801

[B64] Mahajan P. Uddin S. Hajati F. Moni M. A. (2023). Ensemble learning for disease prediction: a review. Healthcare (Basel Switzerland) 11. doi: 10.3390/healthcare11121808 37372925 PMC10298658

[B65] Mahmood S. A. Khalaf A. A. Hamadi S. S. (2025). Basrah score: a novel machine learning-based score for differentiating iron deficiency anemia and beta thalassemia trait using RBC indices. Front. Big Data 8, 1634133. doi: 10.3389/fdata.2025.1634133 40831588 PMC12358405

[B66] Martono Fatmawati F. Mulyanti S. (2023). Risk factors associated with the severity of COVID-19. Malaysian J. Med. Sci. MJMS 30, 84–92. doi: 10.21315/mjms2023.30.3.7 37425387 PMC10325129

[B67] Mendhi S. Sawarkar K. Shete A. Vinchurkar K. Mali S. S. Singh S. . (2025). Smart healthcare: artificial intelligences impact on drug development and patient care. Intelligent Pharm. 3, 225–234. doi: 10.1016/j.ipha.2025.01.003 38826717

[B68] Ministry of Health of U (2020a). “ Protocol "Provision of medical assistance for the treatment of coronavirus disease (COVID-19),” ( Ministry of Health of Ukraine, Kyiv).

[B69] Ministry of Health of U (2020b). “ Medical care standards "Coronavirus disease (COVID-19),” ( Ministry of Health of Ukraine, Kyiv).

[B70] Montesinos López O. A. Montesinos López A. Crossa J. (2022). “ Overfitting, model tuning, and evaluation of prediction performance,” in Multivariate Statistical Machine Learning Methods for Genomic Prediction ( Springer International Publishing, Cham), 109–139. 36103587

[B71] Montiel V. Lobysheva I. Gérard L. Vermeersch M. Perez-Morga D. Castelein T. . (2022). Oxidative stress-induced endothelial dysfunction and decreased vascular nitric oxide in COVID-19 patients. EBioMedicine 77, 103893. doi: 10.1016/j.ebiom.2022.103893 35219085 PMC8865837

[B72] Muhammad I. Naeem A. Haroon A. Faheem M. Yousuf Iqbal B. Umair A. (2024). Predictive modeling of chronic kidney disease using extra tree classifier: a comparative analysis with traditional methods. J. Computing Biomed. Inf. 6, 261–271. doi: 10.29309/tpmj/2015.22.09.1057 20417735

[B73] Nour T. Y. Altintaş K. H. (2023). Effect of the COVID-19 pandemic on obesity and it is risk factors: a systematic review. BMC Public Health 23, 1018. doi: 10.1186/s12889-023-15833-2 37254139 PMC10227822

[B74] Petakh P. Griga V. Mohammed I. B. Loshak K. Poliak I. Kamyshnyiy A. (2022). Effects of metformin, insulin on hematological parameters of COVID-19 patients with type 2 diabetes. Med. Arch. (Sarajevo Bosnia Herzegovina) 76, 329–332. doi: 10.5455/medarh.2022.76.329-332 36545453 PMC9760238

[B75] Powell T. R. Hotopf M. Hatch S. L. Breen G. Duarte R. R. R. Nixon D. F. (2021). Genetic risk for severe COVID-19 correlates with lower inflammatory marker levels in a SARS-CoV-2-negative cohort. Clin. Trans. Immunol. 10, e1292. doi: 10.1002/cti2.1292 34141432 PMC8180242

[B76] Rabbani S. A. El-Tanani M. Sharma S. Rabbani S. S. El-Tanani Y. Kumar R. . (2025). Generative artificial intelligence in healthcare: applications, implementation challenges, and future directions. BioMedInformatics 5, 37. doi: 10.3390/biomedinformatics5030037 30654563

[B77] Rahmani A. M. Yousefpoor E. Yousefpoor M. S. Mehmood Z. Haider A. Hosseinzadeh M. . (2021). Machine learning (ML) in medicine: review, applications, and challenges. Mathematics 9, 2970. doi: 10.3390/math9222970 30654563

[B78] Rahnavard A. Mann B. Giri A. Chatterjee R. Crandall K. A. (2022). Metabolite, protein, and tissue dysfunction associated with COVID-19 disease severity. Sci. Rep. 12, 12204. doi: 10.1038/s41598-022-16396-9 35842456 PMC9288092

[B79] Rane N. Choudhary S. Rane J. (2024). Ensemble deep learning and machine learning: applications, opportunities, challenges, and future directions 18–41. doi: 10.2139/ssrn.4849885

[B80] Rani S. Kumar R. Panda B. S. Kumar R. Muften N. F. Abass M. A. . (2025). Machine learning-powered smart healthcare systems in the era of big data: applications, diagnostic insights, challenges, and ethical implications. Diagnostics 15, 1914. doi: 10.3390/diagnostics15151914 40804880 PMC12346079

[B81] Rattan P. Penrice D. D. Simonetto D. A. (2022). Artificial intelligence and machine learning: what you always wanted to know but were afraid to ask. Gastro Hep Adv. 1, 70–78. doi: 10.1016/j.gastha.2021.11.001 39129929 PMC11307451

[B82] Repchuk Y. Sydorchuk L. Andrii S. Fedonyuk L. Kamyshnyi O. Korovenkova O. . (2021). Linkage of blood pressure, obesity and diabetes mellitus with angiotensinogen gene (AGT 704T>C/rs699) polymorphism in hypertensive patients. Bratislava Med. J. 122, 715–720. doi: 10.4149/bll_2021_114 34570572

[B83] Schoeni R. F. Wiemers E. E. Seltzer J. A. Langa K. M. (2021). Association between risk factors for complications from COVID-19, perceived chances of infection and complications, and protective behavior in the US. JAMA Netw. Open 4, e213984. doi: 10.1001/jamanetworkopen.2021.3984 33787906 PMC8013830

[B84] Schwab P. DuMont Schütte A. Dietz B. Bauer S. (2020). Clinical predictive models for COVID-19: systematic study. J. Med. Internet Res. 22, e21439. doi: 10.2196/21439 32976111 PMC7541040

[B85] Semianiv M. Sydorchuk L. Fedonyuk L. Nebesna Z. Kamyshnyi O. Andrii S. . (2021). Rom J Diabetes Nutr Metab Dis metabolic and hormonal prognostic markers of essential arterial hypertension considering the genes polymorphism AGTR1 (rs5186) and VDR (rs2228570). Rom. J. Diabetes Nutr. Metab. Dis. 28, 284–291.

[B86] Sethi S. Shakyawar S. Reddy A. S. Patel J. C. Guda C. (2024). A machine learning model for the prediction of COVID-19 severity using RNA-Seq, clinical, and co-morbidity data. Diagnostics (Basel Switzerland) 14. doi: 10.3390/diagnostics14121284 38928699 PMC11202902

[B87] Shah P. Shukla M. Dholakia N. H. Gupta H. (2025). Predicting cardiovascular risk with hybrid ensemble learning and explainable AI. Sci. Rep. 15, 17927. doi: 10.1038/s41598-025-01650-7 40410273 PMC12102235

[B88] Sokolenko M. O. Sydorchuk L. P. Sokolenko L. S. Sokolenko A. A. (2024). General immunologic reactivity of patients with COVID-19 and its relation to gene polymorphism, severity of clinical course of the disease and combination with comorbidities. Medicni Perspektivi 29, 108–117. doi: 10.26641/2307-0404.2024.3.313570

[B89] Sokolenko M. Sydorchuk L. Sokolenko A. Sydorchuk R. Kamyshna I. Sydorchuk A. . (2025). Antiviral intervention of COVID-19: linkage of disease severity with genetic markers FGB (rs1800790), NOS3 (rs2070744) and TMPRSS2 (rs12329760). Viruses 17, 792. doi: 10.3390/v17060792 40573382 PMC12197352

[B90] Srivastava S. Dutta A. Guha D. Khatua D. Prasad D. K. Sekh A. A. (2026). Exploring gallbladder cancer prognosis using machine learning and explainable AI. Discover Computing 29, 10. doi: 10.1007/s10791-025-09893-8 30311153

[B91] Stec-Martyna E. Wojtczak K. Nowak D. Stawski R. (2025). Battle of the biomarkers of systemic inflammation. Biology 14, 438. doi: 10.3390/biology14040438 40282303 PMC12024891

[B92] Stojanovic M. Djuric M. Nenadic I. Bojic S. Andrijevic A. Popovic A. . (2026). Vascular complications of long COVID—from endothelial dysfunction to systemic thrombosis: a systematic review. Int. J. Mol. Sci. 27, 433. doi: 10.3390/ijms27010433 41516301 PMC12786942

[B93] Sydorchuk L. Gumennyi R. Škoda M. Sydorchuk A. Vyklyuk Y. Batih I. . (2026). Rigorous management of prediction and classifying of wound healing results in plastic and reconstructive surgery based on machine learning models. Computation 14, 156. doi: 10.3390/computation14070156 30654563

[B94] Sydorchuk L. Sokolenko M. Škoda M. Lajcin D. Vyklyuk Y. Sydorchuk R. . (2025). Management of severe COVID-19 diagnosis using machine learning. Computation 13, 238. doi: 10.3390/computation13100238 30654563

[B95] Sydorchuk A. Sydorchuk L. Gutnitska A. Margaryan A. Dzhuryak V. Sydorchuk R. . (2023a). Linkage of metabolic disorders, endothelial dysfunction and NOS3 (rs2070744) and GNB3 (rs5443) genes polymorphisms in hypertensive patients. Biointerface Res. Appl. Chem. Open-Access J. 13, 1–12.

[B96] Sydorchuk A. Sydorchuk L. Gutnitska A. Vasyuk V. Tkachuk O. Dzhuryak V. . (2023b). The role of NOS3 (rs2070744) and GNB3 (rs5443) genes' polymorphisms in endothelial dysfunction pathway and carotid intima-media thickness in hypertensive patients. Gen. Physiol. Biophys. 42, 179–190. doi: 10.4149/gpb_2022060 36896947

[B97] Tang N. Li D. Wang X. Sun Z. (2020). Abnormal coagulation parameters are associated with poor prognosis in patients with novel coronavirus pneumonia. J. Thromb. Haemostasis JTH 18, 844–847. doi: 10.1111/jth.14768 32073213 PMC7166509

[B98] Tang X. Tang M. Liu W. Cui S. (2026). Explainable machine learning for stroke risk prediction: a comparative study with SHAP-based interpretation. Front. Neurol. 16, 1716984. doi: 10.3389/fneur.2025.1716984 41602961 PMC12832496

[B99] Teng Z. Wang H. Guo F. Han Z. Wang Y. (2023). eNOS polymorphisms on male infertility: an updated systematic review and meta-analysis. Medicine 102, e33993. doi: 10.1097/md.0000000000033993 37327284 PMC10270503

[B100] Thachil J. (2020). The protective rather than prothrombotic fibrinogen in COVID-19 and other inflammatory states. J. Thromb. Haemostasis JTH 18, 1849–1852. doi: 10.1111/jth.14942 32588535 PMC7300603

[B101] Tulu T. W. Wan T. K. Chan C. L. Wu C. H. Woo P. Y. M. Tseng C. Z. S. . (2023). Machine learning-based prediction of COVID-19 mortality using immunological and metabolic biomarkers. BMC Digital Health 1, 6. doi: 10.2139/ssrn.4090855 38014372 PMC9896457

[B102] Ushasree D. Praveen Krishna A. V. Mallikarjuna Rao C. (2024). Enhanced stroke prediction using stacking methodology (ESPESM) in intelligent sensors for aiding preemptive clinical diagnosis of brain stroke. Measurement: Sensors 33, 101108. doi: 10.1016/j.measen.2024.101108 38826717

[B103] Vabalas A. Gowen E. Poliakoff E. Casson A. J. (2019). Machine learning algorithm validation with a limited sample size. PloS One 14, e0224365. doi: 10.1371/journal.pone.0224365 31697686 PMC6837442

[B104] Vitello G. A. Federico C. Bruno F. Vinci M. Musumeci A. Ragalmuto A. . (2022). Allelic variations in the human genes TMPRSS2 and CCR5, and the resistance to viral infection by SARS-CoV-2. Int. J. Mol. Sci. 23, 9171. doi: 10.3390/ijms23169171 36012436 PMC9409186

[B105] Wang D. Hu B. Hu C. Zhu F. Liu X. Zhang J. . (2020). Clinical characteristics of 138 hospitalized patients with 2019 novel coronavirus-infected pneumonia in Wuhan, China. Jama 323, 1061–1069. doi: 10.1001/jama.2020.1585 32031570 PMC7042881

[B106] Wang G. Liu J. Xu R. Liu X. (2022). Elevated plasma D-dimer levels are associated with the poor prognosis of critically ill children. Front. Pediatr. 10, 1001893. doi: 10.3389/fped.2022.1001893 36210933 PMC9537732

[B107] Wolff D. Nee S. Hickey N. S. Marschollek M. (2021). Risk factors for Covid-19 severity and fatality: a structured literature review. Infection 49, 15–28. doi: 10.1007/s15010-020-01509-1 32860214 PMC7453858

[B108] Wynants L. Van Calster B. Collins G. S. Riley R. D. Heinze G. Schuit E. . (2020). Prediction models for diagnosis and prognosis of covid-19: systematic review and critical appraisal. BMJ (Clinical Res. Ed) 369, m1328. doi: 10.1136/bmj.m1328 32265220 PMC7222643

[B109] Xu X. (2026). Machine learning-driven personalized risk prediction: developing an explainable sarcopenia model for older European adults with arthritis. J. Clin. Med. 15. doi: 10.3390/jcm15031022 41682702 PMC12897810

[B110] Yaghoobi A. Lord J. S. Rezaiezadeh J. S. Yekaninejad M. S. Amini M. Izadi P. (2023). TMPRSS2 polymorphism (rs12329760) and the severity of the COVID-19 in Iranian population. PloS One 18, e0281750. doi: 10.1371/journal.pone.0281750 36795725 PMC9934348

[B111] Yang Z. Xu Q. Bao S. Cao X. Huang Q. (2022). Learning with multiclass AUC: theory and algorithms. IEEE Trans. Pattern Anal. Mach. Intell. 44, 7747–7763. doi: 10.1109/tpami.2021.3101125 34329155

[B112] Yates L. Aandahl Z. Richards S. Brook B. (2023). Cross validation for model selection: a review with examples from ecology. Ecol. Monogr. 93. doi: 10.1002/ecm.1557 41531421

[B113] Zafar M. M. Khan Z. A. Javaid N. Aslam M. Alrajeh N. (2025). From data to diagnosis: a novel deep learning model for early and accurate diabetes prediction. Healthcare 13, 2138. doi: 10.3390/healthcare13172138 40941490 PMC12427916

[B114] Zhang J. J. Dong X. Liu G. H. Gao Y. D. (2023). Risk and protective factors for COVID-19 morbidity, severity, and mortality. Clin. Rev. Allergy Immunol. 64, 90–107. doi: 10.1007/s12016-022-08921-5 35044620 PMC8767775

[B115] Zsichla L. Müller V. (2023). Risk factors of severe COVID-19: a review of host, viral and environmental factors. Viruses 15, 175. doi: 10.3390/v15010175 36680215 PMC9863423

